# Supplementary material for: Molecular Characterization of Multidrug-Resistant and Hypervirulent New Delhi Metallo-Beta-Lactamase Klebsiella pneumoniae in Lazio, Italy: A Five-Year Retrospective Study
Source: Antibiotics (Basel). 2024 Nov 5;13(11):1045. doi: 10.3390/antibiotics13111045 (PMC11590914; doi:10.3390/antibiotics13111045)
Supplement: Supplementary file 1 [file antibiotics-13-01045-s001.zip › Supplementary Figure S1.pdf]

Figure S1. Phylogenetic Tree relating to the most represented [1]CTs.

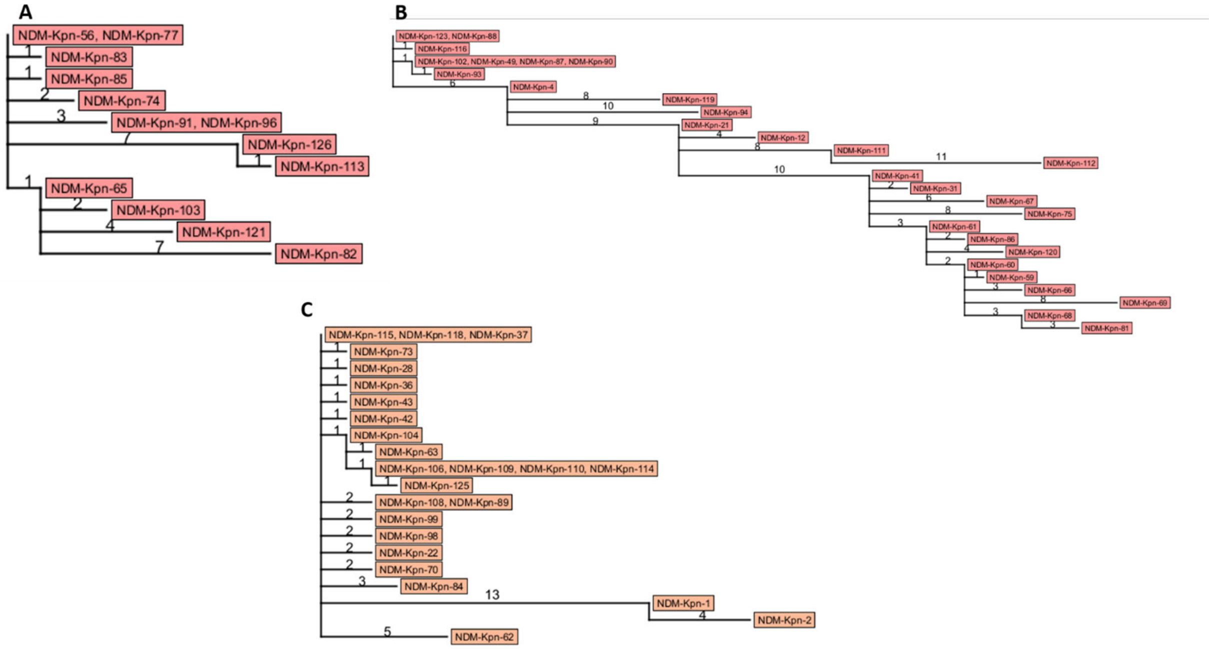

A. CT1:

NDM-Kpn-56: *bla*$_{\text{NDM-1}}$, *bla*$_{\text{OXA-48}}$, *bla*$_{\text{CTX-M-1}}$, *ybt10, ICEKp4*, virulence score 1, [1]H-1, 2023

NDM-Kpn-77: *bla*$_{\text{NDM-1}}$, *bla*$_{\text{OXA-48}}$, *bla*$_{\text{CTX-M-15}}$, *ybt10, ICEKp4*, virulence score 1, H-1, 2023

NDM-Kpn-83: *bla*$_{\text{NDM-1}}$, *bla*$_{\text{OXA-48}}$, *bla*$_{\text{CTX-M-15}}$, *ybt1, ICEKp4*, virulence score 1, H-6, 2023

NDM-Kpn-85: *bla*$_{\text{NDM-1}}$, *bla*$_{\text{OXA-48}}$, *bla*$_{\text{CTX-M-15}}$, *ybt1, ICEKp4*, virulence score 1, H-6, 2023

NDM-Kpn-74: *bla*$_{\text{NDM-1}}$, *bla*$_{\text{OXA-48}}$, *bla*$_{\text{CTX-M-15}}$, *ybt10, ICEKp4*, virulence score 1, H-6, 2023

NDM-Kpn-91: *bla*$_{\text{NDM-1}}$, *bla*$_{\text{OXA-48}}$, *bla*$_{\text{CTX-M-15}}$, *ybt1, ICEKp4*, virulence score 1, H-16, 2023

NDM-Kpn-96: *bla*$_{\text{NDM-1}}$, *bla*$_{\text{OXA-48}}$, *bla*$_{\text{CTX-M-15}}$, *ybt1, ICEKp4*, virulence score 1, H-16, 2023

NDM-Kpn-126: *bla*$_{\text{NDM-1}}$, *bla*$_{\text{OXA-48}}$, *bla*$_{\text{CTX-M-15}}$, *ybt1, ICEKp4*, virulence score 1, H-7, 2023

NDM-Kpn-113: *bla*$_{\text{NDM-1}}$, *bla*$_{\text{OXA-48}}$, *bla*$_{\text{CTX-M-15}}$, *ybt1, ICEKp4*, virulence score 1, H-7, 2023

NDM-Kpn-65: *bla*$_{\text{NDM-1}}$, *bla*$_{\text{OXA-48}}$, *bla*$_{\text{CTX-M-15}}$, *ybt10, ICEKp4*, virulence score 1, H-1, 2023

NDM-Kpn-103: *bla*$_{\text{NDM-1}}$, *bla*$_{\text{OXA-48}}$, *bla*$_{\text{CTX-M-15}}$, *ybt1, ICEKp4*, virulence score 1, H-1, 2023

NDM-Kpn-121: *bla*$_{\text{NDM-1}}$, *bla*$_{\text{OXA-48}}$, *bla*$_{\text{CTX-M-15}}$, *ybt1, ICEKp4*, virulence score 1, H-4, 2023

NDM-Kpn-82: *bla*$_{\text{NDM-1}}$, *bla*$_{\text{OXA-48}}$, *bla*$_{\text{CTX-M-15}}$, *ybt1, ICEKp4*, virulence score 1, H-5, 2023

B. CT3:

NDM-Kpn-123: *bla*$_{\text{NDM-1}}$, *bla*$_{\text{VIM-1}}$, *bla*$_{\text{CTX-M-15}}$, *ybt*9, *ICEKp*3, virulence score 1, H-2, 2023

NDM-Kpn-88: *bla*$_{\text{NDM-1}}$, *bla*$_{\text{VIM-1}}$, *bla*$_{\text{CTX-M-15}}$, *ybt*9, *ICEKp*3, virulence score 1, H-2, 2023

NDM-Kpn-116: *bla*$_{\text{NDM-1}}$, *bla*$_{\text{VIM-1}}$, *bla*$_{\text{CTX-M-15}}$, *ybt9*, *ICEKp3*, virulence score 1, H-7, 2023

NDM-Kpn-102: *bla*$_{\text{NDM-1}}$, *bla*$_{\text{VIM-1}}$, *bla*$_{\text{CTX-M-15}}$, *ybt9*, *ICEKp3*, virulence score 1, H-2, 2023

NDM-Kpn-49: *bla*$_{\text{NDM-1}}$, *bla*$_{\text{CTX-M-15}}$, *ybt9*, *ICEKp3*, virulence score 1, H-4, 2022

NDM-Kpn-87: *bla*$_{\text{NDM-1}}$, *bla*$_{\text{VIM-1}}$, *bla*$_{\text{CTX-M-15}}$, *ybt9*, *ICEKp3*, virulence score 1, H-2, 2023

NDM-Kpn-90: *bla*$_{\text{NDM-1}}$, *bla*$_{\text{VIM-1}}$, *bla*$_{\text{CTX-M-15}}$, *ybt9*, *ICEKp3*, virulence score 1, H-1, 2023

NDM-Kpn-93: *bla*$_{\text{NDM-1}}$, *bla*$_{\text{VIM-1}}$, *bla*$_{\text{CTX-M-15}}$, *ybt9*, *ICEKp3*, virulence score 1, H-1, 2023

NDM-Kpn-4: *bla*$_{\text{NDM-1}}$, *bla*$_{\text{CTX-M-15}}$, *ybt9*, *ICEKp3*, *iuc1*, *rmp*, virulence score 4, H-3, 2019

NDM-Kpn-119: *bla*$_{\text{NDM-1}}$, *bla*$_{\text{CTX-M-15}}$, *ybt9*, *ICEKp3*, *iuc1*, *rmp1*, virulence score 4, H-18, 2023

NDM-Kpn-94: *bla*$_{\text{NDM-1}}$, *bla*$_{\text{CTX-M-15}}$, *ybt9*, *ICEKp3*, *iuc1*, *rmp1*, *KpVP-1*, virulence score 4, H-3, 2023

NDM-Kpn-21: *bla*$_{\text{NDM-1}}$, *bla*$_{\text{CTX-M-15}}$, *ybt9*, *ICEKp3*, *iuc1*, *rmp1*, *KpVP*-1, virulence score 4, H-5, 2021

NDM-Kpn-12: *bla*$_{\text{NDM-1}}$, *bla*$_{\text{CTX-M-15}}$, *ybt9, ICEKp3, iuc1*, *rmp*, virulence score 4, H-8, 2020

NDM-Kpn-111: *bla*$_{\text{NDM-5}}$, *bla*$_{\text{CTX-M-15}}$, *ybt9*, *ICEKp3*, virulence score 1, H-9, 2023

NDM-Kpn-112: *bla*$_{\text{NDM-5}}$, *bla*$_{\text{CMY-2}}$, *bla*$_{\text{CTX-M-15}}$, *ybt9*, *ICEKp3*, *iuc1*, *rmp1*, *KpVP*-1, virulence score 4, H-7, 2023

NDM-Kpn-41: *bla*$_{\text{NDM-1}}$, *bla*$_{\text{CTX-M-15}}$, *ybt9*, *ICEKp3*, *iuc1*, *rmp1*, *KpVP*-1, virulence score 4, H-2, 2022

NDM-Kpn-31: *bla*$_{\text{NDM-1}}$, *bla*$_{\text{CTX-M-15}}$, *ybt9*, *ICEKp3*, *iuc1*, *rmp1*, *KpVP*-1, virulence score 4, H-1, 2021

NDM-Kpn-67: *bla*$_{\text{NDM-1}}$, *bla*$_{\text{CTX-M-15}}$, *ybt9*, *ICEKp3*, *iuc1*, *rmp1*, *KpVP*-1, virulence score 4, H-7, 2023

NDM-Kpn-75: *bla*$_{\text{NDM-1}}$, *bla*$_{\text{CTX-M-15}}$, *ybt9*, *ICEKp3*, virulence score 1, H-1, 2023

NDM-Kpn-61: *bla*$_{\text{NDM-1}}$, *bla*$_{\text{CTX-M-15}}$, *ybt9*, *ICEKp3*, *iuc1*, *rmp1*, *KpVP*-1, virulence score 4, H-8, 2023

NDM-Kpn-86: *bla*$_{\text{NDM-1}}$, *bla*$_{\text{CTX-M-15}}$, *ybt9*, *ICEKp3*, *iuc1*, *rmp1*, *KpVP*-1, virulence score 4, H-4, 2023

NDM-Kpn-120: *bla*$_{\text{NDM-1}}$, *bla*$_{\text{CTX-M-15}}$, *ybt9*, *ICEKp3*, *iuc1*, *rmp1*, *KpVP*-1, virulence score 4, H-1, 2023

NDM-Kpn-60: *bla*$_{\text{NDM-1}}$, *bla*$_{\text{CTX-M-15}}$, *ybt9*, *ICEKp3*, *iuc1*, *rmp1*, *KpVP*-1, virulence score 4, H-11, 2023

NDM-Kpn-59: *bla*$_{\text{NDM-1}}$, *bla*$_{\text{CTX-M-15}}$, *ybt9*, *ICEKp3*, *iuc1*, *rmp1*, *KpVP*-1, virulence score 4, H-12, 2023

NDM-Kpn-66: *bla*$_{\text{NDM-1}}$, *bla*$_{\text{CTX-M-15}}$, *ybt10, ICEKp4*, virulence score 1, H-1, 2023

NDM-Kpn-69: *bla*$_{\text{NDM-1}}$, *bla*$_{\text{CTX-M-15}}$, *ybt9*, *ICEKp3*, *iuc1*, *rmp1*, *KpVP*-1, virulence score 4, H-7, 2023

NDM-Kpn-68: *bla*$_{\text{NDM-1}}$, *bla*$_{\text{CTX-M-1}}$, *ybt9*, *ICEKp3*, *iuc1*, *rmp1*, *KpVP*-1, virulence score 4, H-5, 2023

NDM-Kpn-81: *bla*$_{\text{NDM-1}}$, *bla*$_{\text{CTX-M-15}}$, *ybt9*, *ICEKp3*, *iuc1*, *rmp1*, *KpVP*-1, virulence score 4, H-17, 2023

C. CT10:

NDM-Kpn-115: *bla*$_{\text{NDM-1}}$, *bla*$_{\text{CTX-M-15}}$, *ybt15*, *ICEKp11*, virulence score 1, H-7, 2023

NDM-Kpn-118: *bla*$_{\text{NDM-1}}$, *bla*$_{\text{CTX-M-15}}$, *ybt15*, *ICEKp11*, virulence score 1, H-1, 2023

NDM-Kpn-37: *bla*$_{\text{NDM-1}}$, *bla*$_{\text{CTX-M-15}}$, *ybt15*, *ICEKp11*, virulence score 1, H-1, 2022

NDM-Kpn-73: *bla*$_{\text{NDM-1}}$, *bla*$_{\text{CTX-M-1}}$, *ybt15*, *ICEKp11*, virulence score 1, H-1, 2022

NDM-Kpn-28: *bla*$_{\text{NDM-1}}$, *bla*$_{\text{CTX-M-15}}$, *ybt15*, *ICEKp11*, virulence score 1, H-1, 2021

NDM-Kpn-36: *bla*$_{\text{NDM-1}}$, *bla*$_{\text{CTX-M-15}}$, *ybt15*, *ICEKp11*, virulence score 1, H-13, 2022

NDM-Kpn-43: *bla*$_{\text{NDM-1}}$, *bla*$_{\text{CTX-M-15}}$, *ybt15*, *ICEKp11*, virulence score 1, H-15, 2022

NDM-Kpn-42: *bla*$_{\text{NDM-1}}$, *bla*$_{\text{CTX-M-15}}$, *ybt15*, *ICEKp11*, virulence score 1, H-1, 2022

NDM-Kpn-104: *bla*$_{\text{NDM-1}}$, *bla*$_{\text{CTX-M-15}}$, *ybt15*, *ICEKp11*, virulence score 1, H-7, 2023

NDM-Kpn-63: *bla*$_{\text{NDM-1}}$, *bla*$_{\text{CTX-M-15}}$, *ybt15*, *ICEKp11*, virulence score 1, H-1, 2023

NDM-Kpn-106: *bla*$_{\text{NDM-1}}$, *bla*$_{\text{CTX-M-15}}$, *ybt15*, *ICEKp11*, virulence score 1, H-7, 2023

NDM-Kpn-109: *bla*$_{\text{NDM-1}}$, *bla*$_{\text{CTX-M-15}}$, *ybt15*, *ICEKp11*, virulence score 1, H-7, 2023

NDM-Kpn-110: *bla*$_{\text{NDM-1}}$, *bla*$_{\text{CTX-M-15}}$, *ybt15*, *ICEKp11*, virulence score 1, H-7, 2023

NDM-Kpn-114: *bla*$_{\text{NDM-1}}$, *bla*$_{\text{CTX-M-15}}$, *ybt15*, *ICEKp11*, virulence score 1, H-7, 2023

NDM-Kpn-125: *bla*$_{\text{NDM-1}}$, *bla*$_{\text{VIM-1}}$, *bla*$_{\text{CTX-M-15}}$, *ybt15*, *ICEKp11*, virulence score 1, H-7, 2023

NDM-Kpn-108: *bla*$_{\text{NDM-1}}$, *bla*$_{\text{CTX-M-15}}$, *ybt15*, *ICEKp11*, virulence score 1, H-7, 2023

NDM-Kpn-89: *bla*$_{\text{NDM-1}}$, *ybt15, ICEKp11*, virulence score 1, H-1, 2023

NDM-Kpn-99: *bla*$_{\text{NDM-1}}$, *bla*$_{\text{CTX-M-15}}$, *ybt15, ICEKp11*, virulence score 1, H-1, 2023

NDM-Kpn-98: *bla*$_{\text{NDM-1}}$, *bla*$_{\text{CTX-M-15}}$, *ybt15, ICEKp11*, virulence score 1, H-2, 2023

NDM-Kpn-22: *bla*$_{\text{NDM-1}}$, *bla*$_{\text{CTX-M-15}}$, *ybt15, ICEKp11*, virulence score 1, H-6, 2022

NDM-Kpn-70: *bla*$_{\text{NDM-1}}$, *bla*$_{\text{CTX-M-15}}$, *ybt15, ICEKp11*, virulence score 1, H-7, 2023

NDM-Kpn-84: *bla*$_{\text{NDM-1}}$, *bla*$_{\text{CTX-M-15}}$, *ybt15, ICEKp11*, virulence score 1, H-2, 2023

NDM-Kpn-1: *bla*$_{\text{NDM-1}}$, *ybt15, ICEKp11*, virulence score 1, H-4, 2019

NDM-Kpn-2: *bla*$_{\text{NDM-1}}$, *ybt15, ICEKp11*, virulence score 1, H-1, 2019

NDM-Kpn-62: *bla*$_{\text{NDM-1}}$, *bla*$_{\text{KPC-3}}$, *bla*$_{\text{CTX-M-15}}$, *ybt15, ICEKp11*, virulence score 1, H-8, 2023

[1]CTs: Cluster types; [2]H: Hospital
