# Supplementary material for: Molecular Characterization of Multidrug-Resistant and Hypervirulent New Delhi Metallo-Beta-Lactamase Klebsiella pneumoniae in Lazio, Italy: A Five-Year Retrospective Study
Source: Antibiotics (Basel). 2024 Nov 5;13(11):1045. doi: 10.3390/antibiotics13111045 (PMC11590914; doi:10.3390/antibiotics13111045)
Supplement: Supplementary file 1 [file antibiotics-13-01045-s001.zip › Supplementary Table S1.pdf]

Supplementary Table S1. Antimicrobial susceptibility results for NDM-Kpn clinical isolates

Strain	Date	Sample	Antibiotic MIC values (mg/L)[1]									
			**CZA**	**ETP**	**IPM**	**MEM**	**GEN**	**AMK**	**CIP**	**TZP**	**SXT**	**COL**
**NDM-Kpn-1**	22/05/19	Rectal swab	>256, R	>1, R	>8, R	>8, R	2, S	≤4, S	>1, R	>16/4, R	≤1/19, S	0.125, S
**NDM-Kpn-2**	12/07/19	Rectal swab	>256, R	>1, R	>8, R	>8, R	≤1, S	32, R	>1, R	>16/4, R	>4/76, R	0.5, S
**NDM-Kpn-3**	18/07/19	Wound swab	>256, R	>1, R	>8, R	>8, R	>16, R	32, R	>=4, R	>16/4, R	>4/76, R	0.5, S
**NDM-Kpn-4**	29/07/19	Wound swab	>256, R	>1, R	>8, R	>8, R	>16, R	32, R	>=4, R	>16/4, R	>4/76, R	16, R
**NDM-Kpn-5**	29/08/19	Wound swab	>256, R	>1, R	>8, R	>8, R	2, S	32, R	>=4, R	>16/4, R	>4/76, R	0.5, S
**NDM-Kpn-6**	13/09/19	Blood	>256, R	>1, R	>8, R	>8, R	>16, R	32, R	>=4, R	>16/4, R	>4/76, R	2, S
**NDM-Kpn-7**	27/09/19	Wound swab	>256, R	>1, R	>8, R	>8, R	>4, R	8, S	>1, R	>16/4, R	>4/76, R	0.5, S
**NDM-Kpn-8**	14/11/19	Drainage	>256, R	>1, R	>8, R	>8, R	>16, R	32, R	>=4, R	>16/4, R	>4/76, R	16, R
**NDM-Kpn-9**	18/11/19	Rectal swab	>256, R	>1, R	>8, R	>8, R	>16, R	32, R	>=4, R	>16/4, R	>4/76, R	0,5, S
**NDM-Kpn-10**	18/11/19	Rectal swab	>256, R	>1, R	>8, R	>8, R	>16, R	32, R	>=4, R	>16/4, R	>4/76, R	0,5, S
**NDM-Kpn-11**	30/12/19	Wound swab	>256, R	>1, R	>8, R	>8, R	>16, R	4, S	>=4, R	>16/4, R	>4/76, R	0.5, S
**NDM-Kpn-12**	06/02/20	Blood	>256, R	>1, R	>8, R	>8, R	>16, R	32, R	>=4, R	>16/4, R	>4/76, R	0.5, S
**NDM-Kpn-13**	17/02/20	Femoral cath.	>256, R	>1, R	>8, R	>8, R	>4, R	16, R	>1, R	>16/4, R	≤1/19, S	0.5, S
**NDM-Kpn-14**	22/04/20	Rectal swab	>256, R	>1, R	>8, R	>8, R	≤1, S	≤4, S	>1, R	>16/4, R	>4/76, R	0.25, S
**NDM-Kpn-15**	23/04/20	Blood	>256, R	>1, R	>8, R	>8, R	>4, R	>16, R	>1, R	>16/4, R	≤1/19, S	4, R
**NDM -Kpn-16**	05/05/20	BAL	>256, R	>1, R	>8, R	>8, R	≤1, S	≤4, S	>1, R	>16/4, R	>4/76, R	0.25, S
**NDM -Kpn-17**	30/06/20	Rectal swab	>256, R	>1, R	>8, R	>8, R	>16, R	8, S	>=4, R	>16/4, R	>4/76, R	0.5, S
**NDM -Kpn-18**	06/10/20	Urine	>256, R	>1, R	>8, R	>8, R	>4, R	>16, R	>1, R	>16/4, R	≤1/19, S	8, R
**NDM -Kpn-19**	11/02/21	Rectal swab	>256, R	>1, R	>8, R	>8, R	>4, R	>16, R	>1, R	>16/4, R	>4/76, R	0.5, S
**NDM -Kpn-20**	19/02/21	Rectal swab	>256, R	>2, R	>8, R	>8, R	>8, R	>16, R	>1, R	>16/4, R	>4/76, R	0.5, S
**NDM -Kpn-21**	03/03/21	Urine	>256, R	>1, R	>8, R	>8, R	>4, R	>16, R	>1, R	>16/4, R	>4/76, R	0.25, S
**NDM -Kpn-22**	08/03/21	Rectal swab	>256, R	>1, R	>8, R	>8, R	>4, R	>16, R	>1, R	>64/4, R	>4/76, R	2, R
**NDM -Kpn-23**	23/03/21	Rectal swab	>256, R	>1, R	>8, R	>8, R	>4, R	>16, R	>1, R	>16/4, R	>4/76, R	4, R
**NDM -Kpn-24**	01/04/21	Drainage	>256, R	>1, R	>8, R	>8, R	>4, R	>16, R	>1, R	>16/4, R	>4/76, R	2, S
**NDM -Kpn-25**	01/04/21	Rectal swab	>256, R	>1, R	>8, R	>8, R	>4, R	>16, R	>1, R	>16/4, R	>4/76, R	4, R
**NDM -Kpn-26**	08/04/21	Rectal swab	>256, R	>1, R	>8, R	>8, R	≤ 1, S	>=4, S	>1, R	>16/4, R	≤ 1/19, S	16, R
**NDM -Kpn-27**	30/04/21	Urine	>256, R	>1, R	>8, R	>8, R	>4, R	>16, R	>1, R	>16/4, R	>4/76, R	16, R
**NMD-Kpn-28**	15/06/21	Rectal swab	>256, R	>1, R	>8, R	>8, R	>4, R	>16, R	>1, R	>16/4, R	>4/76, R	2, R
**NDM -Kpn-29**	17/06/21	Rectal swab	>256, R	>1, R	>8, R	>8, R	>4, R	>16, R	>1, R	>16/4, R	>4/76, R	16, R
**NDM -Kpn-30**	17/09/21	BAL	>256, R	>2, R	>8, R	>8, R	>8, R	>16, R	>1, R	>64/4, R	>4/76, R	0.5, S
**NDM -Kpn-31**	23/11/21	Rectal swab	>256, R	>2, R	>8, R	>8, R	≤ 1, S	>16, R	>1, R	>64/4, R	>4/76, R	0.5, S
**NDM-Kpn-32**	10/03/22	Rectal swab	>256, R	>2, R	>8, R	>8, R	≤ 1, S	>16, R	>1, R	>64/4, R	>4/76, R	0.5, S
**NDM-Kpn-33**	31/03/22	Rectal swab	>256, R	>1, R	>8, R	>8, R	>4, R	>16, R	>1, R	>16/4, R	>4/76, R	1, S
**NDM-Kpn-34**	28/03/22	BAL	>256, R	>1, R	>8, R	>8, R	>4, R	>16, R	>1, R	>16/4, R	>4/76, R	1, S
**NDM-Kpn-35**	15/04/22	Rectal swab	>256, R	>1, R	>8, R	>8, R	>4, R	>16, R	>1, R	>16/4, R	>4/76, R	1, S
**NDM-Kpn-36**	18/05/22	Blood	>256, R	>1, R	>8, R	>8, R	>16, R	>16, R	>=4, R	>64/4, R	≤1/19, S	1, S
**NDM-Kpn-37**	24/05/22	Urine	>256, R	>1, R	>8, R	>8, R	2, S	16, R	>1, R	>16/4, R	>4/76, R	1, S
**NDM-Kpn-38**	13/06/22	BAL	>256, R	>1, R	>8, R	>8, R	>4, R	>16, R	>1, R	>16/4, R	>4/76, R	0.5, S
**NDM-Kpn-39**	30/06/22	Rectal swab	>256, R	>2, R	8, R	>8, R	>8, R	>16, R	>1, R	>64/4, R	>4/76, R	0.5, S
**NDM-Kpn-40**	25/07/22	Blood	>256, R	>1, R	>8, R	>8, R	>4, R	16, R	>1, R	>16/4, R	>4/76, R	1, S
**NDM-Kpn-41**	26/07/22	Blood	>256, R	>1, R	>8, R	>8, R	>8, R	>16, R	>1, R	>64/4, R	>4/76, R	0.5, S
**NDM-Kpn-42**	05/08/22	Rectal swab	>256, R	>1, R	>8, R	>8, R	>4, R	16, R	>1, R	>16/4, R	>4/76, R	1, S
**NDM-Kpn-43**	09/08/22	Blood	>256, R	>1, R	>8, R	>8, R	>4, R	8, S	>1, R	>16/4, R	≤ 1/19, S	0.5, S
**NDM-Kpn-44**	15/08/22	BAL	>256, R	>1, R	>8, R	>8, R	>4, R	>16, R	>1, R	>16/4, R	>4/76, R	16, R
**NDM-Kpn-45**	22/08/22	Rectal swab	>256, R	>2, R	>8, R	>8, R	>8, R	8, S	>1, R	>64/4, R	≤ 1/19, S	0.5, S
**NDM-Kpn-46**	22/08/22	Rectal swab	>256, R	>1, R	>8, R	>8, R	32, R	8, S	>8, R	>64/4, R	≤ 1/19, S	1, S
**NDM-Kpn-47**	22/08/22	Rectal swab	>256, R	>2, R	>8, R	>8, R	>8, R	>16, R	>1, R	>64/4, R	>4/76, R	0.5, S
**NDM-Kpn-48**	25/08/22	Urine	>256, R	>1, R	>8, R	>8, R	>=16, R	16, R	>=4, R	>64/4, R	≤ 1/19, S	0.5, S
**NDM-Kpn-49**	23/08/22	Urine	>256, R	>1, R	>8, R	>8, R	2, S	16, R	>1, R	>16/4, R	≤ 1/19, S	0.25, S

**NDM-Kpn-50**	06/09/22	Rectal swab	>256, R	>2, R	>8, R	>8, R	>8, R	>16, R	>1, R	>64/4, R	>4/76, R	0.5, S
**NDM-Kpn-51**	06/09/22	Rectal swab	>256, R	>2, R	>8, R	>8, R	>8, R	16, R	>1, R	>64/4, R	≤ 1/19, S	0.5, S
**NDM-Kpn-52**	06/09/22	Rectal swab	>256, R	>2, R	>8, R	>8, R	>8, R	>16, R	>1, R	>64/4, R	>4/76, R	0.5, S
**NDM-Kpn-53**	09/09/22	Rectal swab	>256, R	>1, R	>8, R	>8, R	>4, R	>16, R	>1, R	>16/4, R	>4/76, R	1, S
**NDM-Kpn-54**	20/09/22	Rectal swab	>256, R	>1, R	>8, R	>8, R	>4, R	>16, R	>1, R	>16/4, R	≤ 1/19, S	1, S
**NDM-Kpn-55**	25/11/22	Wound swab	>256, R	>1, R	>8, R	>8, R	>8, R	>16, R	>1, R	>64/4, R	>4/76, R	0.5, S
**NDM-Kpn-56**	19/01/23	Rectal swab	>256, R	>1, R	>8, R	>8, R	>4, R	>16, R	>1, R	>16/4, R	>4/76, R	1, S
**NDM-Kpn-57**	02/02/23	Rectal swab	>256, R	>1, R	>8, R	>8, R	>4, R	>16, R	>1, R	>16/4, R	>4/76, R	1, S
**NDM-Kpn-58**	14/02/23	Rectal swab	>256, R	>1, R	>8, R	>8, R	>4, R	>16, R	>1, R	>16/4, R	≤ 1/19, S	1, S
**NDM-Kpn-59**	21/02/23	Blood	>256, R	>1, R	>8, R	>8, R	>4, R	>16, R	>1, R	>64/4, R	>4/76, R	16, R
**NDM-Kpn-60**	23/02/23	Urine	>256, R	>1, R	>8, R	>8, R	>4, R	>16, R	>1, R	>64/4, R	>4/76, R	1, S
**NDM-Kpn-61**	01/03/23	Blood	>256, R	>1, R	>8, R	>8, R	>=16, R	32, R	>=4, R	>64/4, R	>4/76, R	8, R
**NDM-Kpn-62**	01/03/23	Blood	>256, R	>1, R	>8, R	>8, R	>=32, R	16, R	>=8, R	>64/4, R	>4/76, R	1, S
**NDM-Kpn-63**	19/05/23	Urine	>256, R	>1, R	>8, R	>8, R	>4, R	16, R	>1, R	>64/4, R	≤ 1/19, S	0.5, S
**NDM-Kpn-64**	19/05/23	Urethral swab	>256, R	>1, R	>8, R	>8, R	>4, R	>16, R	1, R	>64/4, R	>4/76, R	0.5, S
**NDM-Kpn-65**	23/05/23	Urine	>256, R	>1, R	>8, R	>8, R	>4, R	>16, R	>1, R	>64/4, R	>4/76, R	0.5, S
**NDM-Kpn-66**	29/05/23	Rectal swab	>256, R	>1, R	>8, R	>8, R	>4, R	>16, R	>1, R	>16/4, R	>4/76, R	4, R
**NDM-Kpn-67**	29/05/23	Urine	>256, R	>1, R	>8, R	>8, R	>4, R	>16, R	>1, R	>16/4, R	>4/76, R	1, S
**NDM-Kpn-68**	29/05/23	Urine	>256, R	>1, R	>8, R	>8, R	>4, R	>16, R	>1, R	>16/4, R	>4/76, R	0.25, S
**NDM-Kpn-69**	29/05/23	Urine	>256, R	>1, R	>8, R	>8, R	2, S	8, S	>1, R	>16/4, R	>4/76, R	4, R
**NDM-Kpn-70**	29/05/23	Rectal swab	>256, R	>1, R	>8, R	>8, R	>4, R	>8, R	>1, R	>16/4, R	≤ 1/19, S	1, S
**NDM-Kpn-71**	30/05/23	Rectal swab	>256, R	>1, R	>8, R	>8, R	>4, R	>16, R	1, R	>16/4, R	>4/76, R	1, S
**NDM-Kpn-72**	06/06/23	Rectal swab	>256, R	>2, R	>8, R	>8, R	>8, R	>16, R	>1, R	>128, R	>4/76, R	0.5, S
**NDM-Kpn-73**	09/06/23	Rectal swab	>256, R	>1, R	>8, R	>8, R	>4, R	16, R	>1, R	>16/4, R	>4/76, R	1, S
**NDM-Kpn-74**	19/06/23	Rectal swab	>256, R	>1, R	>8, R	>8, R	>4, R	>16, R	>1, R	>64/4, R	>4/76, R	1, S
**NDM-Kpn-75**	04/07/23	Rectal swab	>256, R	>1, R	>8, R	>8, R	2, S	16, R	1, R	>64/4, R	≤ 1/19, S	1, S
**NDM-Kpn-76**	04/07/23	Rectal swab	>256, R	>1, R	>8, R	>8, R	>4, R	>16, R	>1, R	>16/4, R	>4/76, R	1, S
**NDM-Kpn-77**	04/07/23	Rectal swab	>256, R	>1, R	>8, R	>8, R	>4, R	>16, R	>1, R	>16/4, R	>4/76, R	1, S
**NDM-Kpn-78**	11/07/23	Rectal swab	>256, R	>1, R	>8, R	>8, R	≤1, S	≤4, S	>1, R	>16/4, R	>4/76, R	1, S
**NDM-Kpn-79**	26/07/23	Urine	>256, R	>1, R	>8, R	>8, R	>4, R	>16, R	>1, R	64/4, R	>4/76, R	1, S
**NDM-Kpn-80**	02/08/23	Urine	>256, R	>1, R	>8, R	>8, R	>4, R	>16, R	>1, R	>16/4, R	>4/76, R	1, S
**NDM-Kpn-81**	02/08/23	Rectal swab	>8/4, R	>1, R	>8, R	>8, R	>=16, R	>16, R	>=4, R	>64/4, R	>4/76, R	1, S
**NDM-Kpn-82**	12/08/23	Rectal swab	>8/4, R	>1, R	>8, R	>8, R	>4, R	>16, R	>1, R	>16/4, R	>4/76, R	1, S
**NDM-Kpn-83**	16/08/23	Rectal swab	>8/4, R	>1, R	>8, R	>8, R	>4, R	>16, R	>1, R	>64/4, R	>4/76, R	1, S
**NDM-Kpn-84**	22/08/23	Urine	>8/4, R	>1, R	>8, R	>8, R	32, R	8, S	>8, R	>64/4, R	≤ 1/19, S	1, S
**NDM-Kpn-85**	22/08/23	Rectal swab	>8/4, R	>1, R	>8, R	>8, R	>4, R	>16, R	>1, R	>16/4, R	>4/76, R	1, S
**NDM-Kpn-86**	04/09/23	Rectal swab	>8/4, R	>1, R	>8, R	>8, R	>4, R	>16, R	>1, R	>64/4, R	>4/76, R	4, R
**NDM-Kpn-87**	08/09/23	Vaginal swab	>8/4, R	>1, R	>8, R	>8, R	1, S	8, S	>8, R	>64/4, R	>4/76, R	1, S
**NDM-Kpn-88**	08/09/23	Bronchial Aspirate	>8/4, R	>1, R	>8, R	>8, R	1, S	>16, R	>8, R	>64/4, R	>4/76, R	1, S
**NDM-Kpn-89**	12/09/23	Rectal swab	256/4 R	>1, R	>8, R	>8, R	>4, R	16, R	>1, R	>16/4, R	≤ 1/19, S	1, S
**NDM-Kpn-90**	12/09/23	Rectal swab	256/4 R	>1, R	>8, R	>8, R	2, S	16, R	>1, R	>16/4, R	>4/76, R	1, S
**NDM-Kpn-91**	19/09/23	Urine	>16/4, R	>1, R	>8, R	>8, R	>4, R	>16, R	>1, R	>16/4, R	>4/76, R	1, S
**NDM-Kpn-92**	20/09/23	Blood	>=16, R	>1, R	>8, R	>8, R	>=16, R	>16, R	>1, R	>64/4, R	>4/76, R	1, S
**NDM-Kpn-93**	27/09/23	Urine	>8/4, R	>1, R	>8, R	>8, R	>4, R	16, R	>1, R	>16/4, R	<=1/19, S	0.5, S
**NDM-Kpn-94**	28/09/23	Wound swab	>128, R	>8, R	>8, R	>8, R	>=16, R	32, R	>=4, R	>64/4, R	>=320, R	0.5, S
**NDM-Kpn-95**	03/10/23	Urine	>128, R	>8, R	>8, R	>8, R	32, R	>16, R	1, R	>64/4, R	>4/76, R	1, S
**NDM-Kpn-96**	06/10/23	Urine	>8/4, R	>1, R	>8, R	>8, R	>4, R	>16, R	>1, R	>16/4, R	>4/76, R	1, S
**NDM-Kpn-97**	18/10/23	Blood	>8/4, R	>2, R	>8, R	>8, R	>4, R	>32, R	>1, R	>16/4, R	>4/76, R	1, S
**NDM-Kpn-98**	24/10/23	Urine	>8/4, R	>1, R	>8, R	>8, R	>=16, R	>16, R	>=4, R	>64/4, R	≤ 1/19, S	0.5, S
**NDM-Kpn-99**	10/11/23	Rectal swab	256/4, R	>1, R	>8, R	>8, R	>4, R	>16, R	>1, R	>16/4, R	≤ 1/19, S	1, S
**NDM-Kpn-100**	15/11/23	Rectal swab	>8/4, R	>1, R	>8, R	>8, R	1, S	≤1, S	>1, R	>64/4, R	>4/76, R	1, S

**NDM-Kpn-101**	15/11/23	Bronchial Aspirate	>8/4, R	>1, R	>8, R	>8, R	>32, R	>32, R	>8, R	>64/4, R	>4/76, R	1, S
**NDM-Kpn-102**	15/11/23	Urine	>8/4, R	>2, R	>8, R	>8, R	<=1, S	8, S	>1, R	>64/4, R	>4/76, R	0.5, S
**NDM-Kpn-103**	24/11/23	Rectal swab	256/4, R	>1, R	>8, R	>8, R	>4, R	>16, R	>1, R	>16/4 R	>4/76, R	1, S
**NDM-Kpn-104**	24/11/23	Urine	>8/4, R	>1, R	>8, R	>8, R	>4, R	16, R	>1, R	>16/4 R	≤ 1/19, S	1, S
**NDM-Kpn-105**	24/11/23	Urine	>8/4, R	>1, R	>8, R	>8, R	>4, R	>16, R	1, R	>16/4 R	>4/76, R	1, S
**NDM-Kpn-106**	24/11/23	Urine	>8/4, R	>1, R	>8, R	>8, R	>4, R	16, R	>1, R	>16/4 R	≤ 1/19, S	1, S
**NDM-Kpn-107**	24/11/23	Rectal swab	>8/4, R	>1, R	>8, R	>8, R	2, S	8, S	>1, R	>16/4 R	≤ 1/19, S	1, S
**NDM-Kpn-108**	24/11/23	Urine	>8/4, R	>1, R	>8, R	>8, R	>4, R	16, R	>1, R	>16/4 R	≤ 1/19, S	1, S
**NDM-Kpn-109**	24/11/23	Urine	>8/4, R	>1, R	>8, R	>8, R	2, S	16, R	>1, R	>16/4, R	≤ 1/19, S	1, S
**NDM-Kpn-110**	24/11/23	Rectal swab	>8/4, R	>1, R	>8, R	>8, R	2, S	16, R	>1, R	>16/4, R	≤ 1/19, S	1, S
**NDM-Kpn-111**	24/11/23	Wound swab	>8/4, R	>1, R	>8, R	>8, R	2, S	16, R	>1, R	>16/4, R	≤ 1/19, S	1, S
**NDM-Kpn-112**	24/11/23	Blood	>8/4, R	>1, R	>8, R	>8, R	>4, R	16, R	>1, R	>16/4, R	>4/76, R	1, S
**NDM-Kpn-113**	24/11/23	Rectal swab	>8/4, R	>1, R	>8, R	>8, R	>4, R	>16, R	>1, R	>16/4, R	>4/76, R	1, S
**NDM-Kpn-114**	24/11/23	Rectal swab	>8/4, R	>1, R	>8, R	>8, R	2, S	>8, R	>1, R	>16/4, R	≤ 1/19, S	1, S
**NDM-Kpn-115**	24/11/23	Rectal swab	>8/4, R	>1, R	>8, R	>8, R	>4, R	16, R	>1, R	>16/4, R	≤ 1/19, S	1, S
**NDM-Kpn-116**	24/11/23	Rectal swab	>8/4, R	>1, R	>8, R	>8, R	2, S	>16, R	>1, R	>64/4, R	>4/76, R	0.5, S
**NDM-Kpn-117**	20/11/23	Urine	>8/4, R	>1, R	>8, R	>8, R	<=1, S	8, S	>1, R	>64/4, R	>4/76, R	0.25, S
**NDM-Kpn-118**	29/11/23	Urine	>8/4, R	>1, R	>8, R	>8, R	>4, R	16, R	>1, R	>16/4, R	>4/76, R	1, S
**NDM-Kpn-119**	01/12/23	Wound swab	>8/4, R	>1, R	>8, R	>8, R	>4, R	>16, R	>1, R	>64/4, R	>4/76, R	0.25, S
**NDM-Kpn-120**	12/12/23	Rectal swab	256/4, R	>1, R	>8, R	>8, R	>4, R	>16, R	>1, R	>16/4, R	>4/76, R	4, R
**NDM-Kpn-121**	07/12/23	Urine	256/4, R	>1, R	>8, R	>8, R	>4, R	>8, R	>1, R	>16/4, R	>4/76, R	1, S
**NDM-Kpn-122**	14/12/23	Rectal swab	>8/4, R	>1, R	>8, R	>8, R	>4, R	>16, R	>1, R	>16/4, R	>4/76, R	1, S
**NDM-Kpn-123**	15/12/23	Rectal swab	>8/4, R	>1, R	>8, R	>8, R	>4, R	>16, R	>8, R	>64/4, R	>4/76, R	1, S
**NDM-Kpn-124**	20/12/23	Urine	>8/4, R	>1, R	>8, R	>8, R	>4, R	>16, R	>1, R	>16/4, R	>4/76, R	1, S
**NDM-Kpn-125**	20/12/23	Urine	>8/4, R	>1, R	>8, R	>8, R	2, S	16, R	>1, R	>16/4, R	≤ 1/19, S	1, S
**NDM-Kpn-126**	20/12/23	Urine	>8/4, R	>1, R	>8, R	>8, R	2, S	>16, R	>1, R	>16/4, R	>4/76, R	1, S

[1]CZA, ceftazidime/avibactam; ERT, ertapenem; IPM, imipenem; MEM, meropenem; GEN, gentamicin; AMK, amikacin; CIP, ciprofloxacin; TZP, piperacillin-tazobactam; SXT, trimethoprim-sulfametossazole; COL, colistin; S, susceptible; R, resistant.
