# Supplementary material for: Molecular Characterization of Multidrug-Resistant and Hypervirulent New Delhi Metallo-Beta-Lactamase Klebsiella pneumoniae in Lazio, Italy: A Five-Year Retrospective Study
Source: Antibiotics (Basel). 2024 Nov 5;13(11):1045. doi: 10.3390/antibiotics13111045 (PMC11590914; doi:10.3390/antibiotics13111045)
Supplement: Supplementary file 1 [file antibiotics-13-01045-s001.zip › Supplementary Table S2.pdf]

Supplementary Table S2. Molecular characterization of NDM-Kpn clinical isolates

Strain	Resistance determinants	
	**Beta-lactams**	**Additional resistance genes**
NDM-Kpn-1	***bla***$_{\textbf{NDM-1}}$, *bla*$_{\text{SHV-107}}$	*aac(6)-Ib, fosA_3, oqxA, oqxB, dfrA14*
NDM-Kpn-2	***bla***$_{\textbf{NDM-1}}$, *bla*$_{\text{SHV-11}}$	*fosA_3, oqxA, oqxB, dfrA14*
NDM-Kpn-3	***bla***$_{\textbf{NDM-1}}$, ***bla***$_{\textbf{OXA-48}}$, ***bla***$_{\textbf{CTX-M-15}}$, ***bla***$_{\textbf{CMY-6}}$, *bla*$_{\text{SHV-28}}$	*aac(6)-Ib, rmtC, fosA_3, oqxA, oqxB, sul1, sul2, dfrA14*
NDM-Kpn-4	***bla***$_{\textbf{NDM-1}}$, ***bla***$_{\textbf{CTX-M-15}}$, *bla*$_{\text{OXA-1}}$, *bla*$_{\text{OXA-9}}$, *bla*$_{\text{SHV-81}}$, *bla*$_{\text{TEM-1}}$	*aph(3')-VI, aac(6')-Ib, aadA1, armA, aac(6')-Ib-cr, fosA_3, fosA_5, msr(E), mph(A), mph(E), catB3, qnrS1, oqxA, oqxB, ARR-3, sul1, sul2, dfrA5*
NDM-Kpn-5	***bla***$_{\textbf{NDM-5}}$, ***bla***$_{\textbf{CTX-M-15}}$, *bla*$_{\text{OXA-9}}$, *bla*$_{\text{SHV-26}}$, *bla*$_{\text{TEM-1}}$	*aph(3')-VI, aac(6')-Ib, aadA1, aph(3')-Ia, armA, fosA_3, fosA_5, msr(E), mph(A), mph(E), catA1, qnrS1, oqxA, oqxB, sul1, sul2, tet(A), dfrA5*
NDM-Kpn-6	***bla***$_{\textbf{NDM-1}}$, ***bla***$_{\textbf{OXA-48}}$, ***bla***$_{\textbf{CTX-M-15}}$, ***bla***$_{\textbf{CMY-6}}$, *bla*$_{\text{SHV-55}}$	*aac(6')-Ib, rmtC ,fosA_3, oqxA, oqxB, sul1, sul2, dfrA14*
NDM-Kpn-7	***bla***$_{\textbf{NDM-1}}$, ***bla***$_{\textbf{CTX-M-15}}$, *bla*$_{\text{OXA-1}}$, *bla*$_{\text{OXA-10}}$, *bla*$_{\text{SHV-28}}$, *bla*$_{\text{TEM-1}}$	*aph(6)-Id, aac(6')-II, aph(3')-VI, aac(3')-II, aac(6')-Ib-cr, aph(3'')-Ib, fosA_3, catB3, qnrB1, qnrS1, oqxA, oqxB, sul1, sul2, tet(A), dfrA14*
NDM-Kpn-8	***bla***$_{\textbf{NDM-1}}$, ***bla***$_{\textbf{OXA-48}}$, ***bla***$_{\textbf{CTX-M-15}}$, ***bla***$_{\textbf{CMY-6}}$, *bla*$_{\text{SHV-106}}$	*aac(6')-Ib, rmtC, fosA_3, oqxA, oqxB, sul1, sul2*
NDM-Kpn-9	***bla***$_{\textbf{NDM-1}}$, ***bla***$_{\textbf{OXA-48}}$, ***bla***$_{\textbf{CTX-M 14b}}$, ***bla***$_{\textbf{CMY-6}}$, *bla*$_{\text{SHV-148}}$, *bla*$_{\text{TEM-1}}$	*aph(6)-Id, aac(3')-IIa, aac(6')-Ib, aph(3')-VI, aph(3'')-Ib, rmtC, mph(A), fosA_3, catA1, oqxA, oqxB, sul1, tet(A)*
NDM-Kpn-10	***bla***$_{\textbf{NDM-1}}$, ***bla***$_{\textbf{OXA-48}}$, ***bla***$_{\textbf{CMY-6}}$, ***bla***$_{\textbf{CTX-M-15}}$, ***bla***$_{\textbf{CTX-M-14b}}$, *bla*$_{\text{SHV-148}}$, *bla*$_{\text{OXA-9}}$, *bla*$_{\text{TEM-1}}$	*aph(6)-Id, aac(6')-Ib, aph(3')-VI, rmtC, mph(A), aac(6')-Ib-cr, aph(3'')-Ib, fosA_3, catA1, qnrS1, oqxA, oqxB, sul1, sul2, tet(A)*
NDM-Kpn-11	***bla***$_{\textbf{NDM-1}}$, ***bla***$_{\textbf{CTX-M-15}}$, ***bla***$_{\textbf{SCO-1}}$, *bla*$_{\text{SHV-155}}$, *bla*$_{\text{OXA-1}}$, *bla*$_{\text{TEM-1}}$	*aph(6)-Id, aac(3')-IIa, addA1, aac(6')-Ib-cr, fosA_3, catB3, qnrB1, oqxA, oqxB, sul1, sul2, tet(A), dfrA15*
NDM-Kpn-12	***bla***$_{\textbf{NDM-1}}$, ***bla***$_{\textbf{CTX-M-15}}$, *bla*$_{\text{OXA-1}}$, *bla*$_{\text{OXA-9}}$, *bla*$_{\text{SHV-11}}$, *bla*$_{\text{TEM-1}}$	*aac(6')-Ib, aph(3')-VI, aph(3')-Ia aac(6')-Ib-cr, aadA1, armA, msr(E), mph(A), mph(E), fosA_5, catB3, qnrS1, oqxA, oqxB, ARR-3, sul1, sul2, dfrA5*
NDM-Kpn-13	***bla***$_{\textbf{NDM-1}}$, ***bla***$_{\textbf{OXA-48}}$, ***bla***$_{\textbf{CTX-M-14b}}$, *bla*$_{\text{SHV-148}}$	*aph(6)-Id, aph(3'')-Ib, rmtF, mph(A), fosA_3, fosA_5, catA1, qnrB1, oqxA, oqxB, AAR-2, tet(A)*
NDM-Kpn-14	***bla***$_{\textbf{NDM-1}}$, ***bla***$_{\textbf{CTX-M-15}}$, *bla*$_{\text{OXA-1}}$, *bla*$_{\text{SHV-11}}$, *bla*$_{\text{TEM-1}}$	*aadA1, aac(6')-Ib-cr, fosA_3, fosA_5, catB3, qnrB1, oqxA, oqxB, sul1, dfrA15*
NDM-Kpn-15	***bla***$_{\textbf{NDM-1}}$, ***bla***$_{\textbf{OXA-48}}$, ***bla***$_{\textbf{CTX-M-14b}}$, *bla*$_{\text{SHV-148}}$	*aph(6)-Id, aph(3'')-Ib, rmtF, mph(A), fosA_3, fosA_5, catA1, qnrB1, oqxA, oqxB, AAR-2, tet(A)*
NDM -Kpn-16	***bla***$_{\textbf{NDM-1}}$, ***bla***$_{\textbf{CTX-M-15}}$, *bla*$_{\text{OXA-1}}$, *bla*$_{\text{SHV-81}}$, *bla*$_{\text{TEM-1}}$	*aadA1, aac(6')-Ib-cr, fosA_3, fosA_5, catB3, qnrB1, oqxA, oqxB, sul1, dfrA1*
NDM -Kpn-17	***bla***$_{\textbf{NDM-1}}$, *bla*$_{\text{OXA-10}}$, *bla*$_{\text{SHV-2}}$, *bla*$_{\text{SHV-187}}$, *bla*$_{\text{TEM-1}}$	*aac(6')-II, aph(3')-VI, acc(3)-II, aadA16, aac(6')-Ib-cr, fosA_3, floR_2, qnrB6, qnrS1, oqxA, oqxB, ARR-3, sul1, sul2, tet(A), tet(D), dfrA27*
NDM -Kpn-18	***bla***$_{\textbf{NDM-1}}$, ***bla***$_{\textbf{OXA-48}}$, ***bla***$_{\textbf{CTX-M-15}}$, *bla*$_{\text{SHV-148}}$	*aac(6')-Ib, aph(3')-Ib, rmtF, strA, strB, mph(A), oqxA, oqxB, fosA_3, fosA_5, catA1, qnrB1, AAR-2, tet(A)*
NDM -Kpn-19	***bla***$_{\textbf{NDM-1}}$, ***bla***$_{\textbf{CTX-M-15}}$, *bla*$_{\text{OXA-1}}$, *bla*$_{\text{TEM-1}}$, *bla*$_{\text{SHV-182}}$	*aph(3')-VI, armA, aac(6')-Ib-cr, qacE, oqxA, oqxB, fosA_3, msr(E), mph(A), mph(E), catB3, catA1, qnrS1, sul1, sul2, tet(A), dfrA5, dfrA1*
NDM -Kpn-20	***bla***$_{\textbf{NDM-1}}$, ***bla***$_{\textbf{CTX-M-15}}$, *bla*$_{\text{OXA-1}}$, *bla*$_{\text{TEM-1}}$, *bla*$_{\text{SHV-182}}$	*aph(3')-VI, armA, aac(6')-Ib-cr, qacE, oqxA, oqxB, fosA_3, msr(E), mph(A), mph(E), catB3, catA1, qnrS1, sul1, sul2, tet(A), dfrA5, dfrA1*
NDM -Kpn-21	***bla***$_{\textbf{NDM-1}}$, ***bla***$_{\textbf{CTX-M-15}}$, *bla*$_{\text{OXA-1}}$, *bla*$_{\text{OXA-9}}$, *bla*$_{\text{TEM-1}}$, *bla*$_{\text{SHV-67}}$	*aph(3')-VI, acc(6')-Ib, ant(3'')-Ia, aph(3')-Ia, armA, aac(6')-Ib-cr, qac(E), oqxA, oqxB, fosA_5, msr(E), mph(E), mph(A), catB3, qnrB1, sul1, sul2, ARR-3, dfrA5*
NDM -Kpn-22	***bla***$_{\textbf{NDM-1}}$, ***bla***$_{\textbf{CTX-M-15}}$, *bla*$_{\text{OXA-1}}$, *bla*$_{\text{SHV-182}}$	*acc(6')-Ib, aac(6')-Ib-cr, aac(3)-IIa, oqxA, oqxB, fosA_3, catB3, dfrA14*
NDM -Kpn-23	***bla***$_{\textbf{NDM-5}}$, ***bla***$_{\textbf{CTX-M-15}}$, *bla*$_{\text{TEM-1}}$, *bla*$_{\text{SHV-11}}$	*ant(3'')-Ia, armA, rmtB, qacE, oqxA, oqxB, fosA_5, erm(B), msr(E), mph(A), mph(E), ere(A), cmlA1, qnrB1, ARR-2, sul1, dfrA12*
NDM -Kpn-24	***bla***$_{\textbf{NDM-1}}$, ***bla***$_{\textbf{CTX-M-15}}$, *bla*$_{\text{OXA-1}}$, *bla*$_{\text{TEM-1}}$, *bla*$_{\text{SHV-182}}$	*aph(3')-VI, armA, aac(6')-Ib-cr, qac(E), oqxA, oqxB, fosA_3, msr(E), mph(A), mph(E), catB3, catA1, qnrS1, sul1, sul2, tet(A), dfrA5, dfrA1*
NDM -Kpn-25	***bla***$_{\textbf{NDM-5}}$, ***bla***$_{\textbf{CTX-M-15}}$, *bla*$_{\text{TEM-1}}$, *bla*$_{\text{SHV-11}}$	*ant(3'')-Ia, aadA2, armA, rmtB, qacE, oqxA, oqxB, fosA_5, erm(B), msr(E), mph(A), mph(E), ere(A), cmlA1, qnrB1, ARR-2, sul1, dfrA12*
NDM -Kpn-26	***bla***$_{\textbf{NDM-5}}$, ***bla***$_{\textbf{CTX-M-15}}$, *bla*$_{\text{TEM-1}}$, *bla*$_{\text{SHV-11}}$	*ant(3'')-Ia, aadA2, armA, rmtB, qacE, oqxA, oqxB, fosA_5, erm(B), msr(E), mph(A), mph(E), ere(A), cmlA1, qnrB1, ARR-2, sul1, dfrA12*
NDM -Kpn-27	***bla***$_{\textbf{NDM-5}}$, ***bla***$_{\textbf{CTX-M-15}}$, *bla*$_{\text{TEM-1}}$, *bla*$_{\text{SHV-11}}$	*ant(3'')-Ia, aadA2, armA, rmtB, qacE, oqxA, oqxB, fosA_5, erm(B), msr(E), mph(A), mph(E), ere(A), cmlA1, qnrB1, ARR-2, sul1, dfrA12*
NMD-Kpn-28	***bla***$_{\textbf{NDM-1}}$, ***bla***$_{\textbf{CTX-M-15}}$, *bla*$_{\text{OXA-1}}$, *bla*$_{\text{TEM-1}}$, *bla*$_{\text{SHV-182}}$	*aac(6')-Ib, aac(6')-Ib-cr, aadA2, aac(3')-IIa, qac(E), oqxA, oqxB, fosA_3, mph(A), catB3, sul1, dfrA12, dfrA14*
NDM -Kpn-29	***bla***$_{\textbf{NDM-5}}$, ***bla***$_{\textbf{CTX-M-15}}$, *bla*$_{\text{TEM-1}}$, *bla*$_{\text{SHV-67}}$	*ant(3'')-Ia, aadA2, armA, rmtB, qacE, oqxA, oqxB, fosA_5, erm(B), msr(E), mph(A), mph(E), ere(A), cmlA1, qnrB1, ARR-2, sul1, dfrA12*
NDM -Kpn-30	***bla***$_{\textbf{NDM-1}}$, ***bla***$_{\textbf{CTX-M-15}}$, *bla*$_{\text{OXA-1}}$, *bla*$_{\text{SHV-182}}$	*aph(3')-VI, armA, aac(6')-Ib-cr, qac(E), oqxA, oqxB, fosA_3, msr(E), mph(A), mph(E), catB3, catA1, qnrS1, sul1, sul2, tet(A), dfrA5, dfrA1*
NDM -Kpn-31	***bla***$_{\textbf{NDM-1}}$, ***bla***$_{\textbf{CTX-M-15}}$, *bla*$_{\text{OXA-1}}$, *bla*$_{\text{OXA-9}}$, *bla*$_{\text{TEM-1}}$, *bla*$_{\text{SHV-11}}$	*aph(6)-Id, aph(3')-VI, acc(2')-Ib, acc(6')-Ib, aadA1, aph(3')-Ia, armA, aac(6')-Ib-cr, qac(E), oqxA, oqxB, fosA_3, fosA_5, msr(E), mph(A), mph(E) erm(37), catB3, qnrB1, sul1, sul2, ARR-3, dfrA5*
NDM-Kpn-32	***bla***$_{\textbf{NDM-1}}$, ***bla***$_{\textbf{CTX-M-15}}$, *bla*$_{\text{SHV-11}}$	*aph(3')-VI, aph(3')-Ia, armA, oqxA, oqxB, fosA_3, fosA_5, msr(E), mph(A), mph(E), qnrS1, sul2,*

NDM-Kpn-33	**bla_{NDM-1}**, **bla_{KPC-3}**, **bla_{CMY-6}**, bla_{OXA-9}, bla_{TEM-1}, $bla_{SHV-182}$	*aph(3’)-Ia, acc(6’)-Ib, aadA2, rmtC, qac(E), oqxA, oqxB, fosA_3, msr(E), mph(A), mph(E), catA1, qnrB1, sul1, dfrA12*
NDM-Kpn-34	**bla_{NDM-1}**, **bla_{KPC-3}**, **bla_{CMY-6}**, bla_{OXA-9}, bla_{TEM-1}, $bla_{SHV-182}$	*aph(3’)-Ia, acc(6’)-Ib3, aadA2, rmtC, qac(E), oqxA, oqxB, fosA_3, msr(E), mph(A), catA1, sul1, dfrA12*
NDM-Kpn-35	**bla_{NDM-1}**, **$bla_{CTX-M-15}$**, bla_{TEM-1}, bla_{SHV-1}	*aph(3’)-VI, armA, aac(6’)-Ib-cr, qac(E), oqxA, oqxB, fosA_3, msr(E), mph(A), mph(E), catB3, catA1, qnrS1, sul1, sul2, dfrA5*
NDM-Kpn-36	**bla_{NDM-1}**, **$bla_{CTX-M-15}$**, bla_{OXA-1}, $bla_{SHV-182}$	*aac(6’)-Ib, aac(6’)-Ib-cr, aac(3’)-IIa, qac(E), oqxA, oqxB, fosA_3, catB3, dfrA14*
NDM-Kpn-37	**bla_{NDM-1}**, **$bla_{CTX-M-15}$**, bla_{OXA-1}, $bla_{SHV-182}$	*aac(6’)-Ib, aac(6’)-Ib-cr, aac(3’)-Ia, qac(E), oqxA, oqxB, fosA_3, catB3, dfrA14*
NDM-Kpn-38	**bla_{NDM-1}**, **bla_{KPC-3}**, bla_{OXA-9}, bla_{TEM-1}, $bla_{SHV-182}$	*aph(3’)-Ia, acc(6’)-Ib3, aadA2, rmtC, qac(E), oqxA, oqxB, fosA_3, mph(A), catA1, sul1, dfrA12*
NDM-Kpn-39	**bla_{NDM-1}**, **$bla_{CTX-M-15}$**, bla_{OXA-1}, $bla_{SHV-182}$	*aac(3’)-IIa, aac(6’)-Ib-cr, aadA2, aph(3’)-Ia, rmtF, qac(E), oqxA, oqxB, fosA_3, mph(A), catB3, qnrB1, ARR-2, sul1, dfrA12*
NDM-Kpn-40	**bla_{NDM-1}**, **$bla_{CTX-M-55}$**, bla_{LAP-2}, bla_{SHV-51}	*aph(3’)-VI, qac(E), oqxA, oqxB, fosA_3, mph(A), catA1, sul1, sul2, qnrS1, dfrA5, tet(A)*
NDM-Kpn-41	**bla_{NDM-1}**, **$bla_{CTX-M-15}$**, bla_{OXA-1}, bla_{OXA-9}, bla_{TEM-1}, bla_{SHV-67}	*aadA1, aph(3’)-VI, armA, aac(6’)-Ib-cr, qac(E), oqxA, oqxB, fosA_5, msr(E), mph(A), mph(E), catB3, qnrS1, AAR-3, sul1, sul2, dfrA5*
NDM-Kpn-42	**bla_{NDM-1}**, **$bla_{CTX-M-15}$**, bla_{OXA-1}, $bla_{SHV-182}$	*aac(3’)-IIa, aac(6’)-Ib-cr, aac(6’)-Ib, oqxA, oqxB, fosA_3, catB3, dfrA14*
NDM-Kpn-43	**bla_{NDM-1}**, **$bla_{CTX-M-15}$**, bla_{OXA-1}, bla_{TEM-1}, bla_{SHV-12}	*ant(2’’)-Ia, aac(6’)-Ib, aac(6’)-Ib-cr, aph(3’)-Ia, oqxA, oqxB, fosA_3, catB3, qnrB19, dfrA14*
NDM-Kpn-44	**bla_{NDM-1}**, **bla_{KPC-3}**, **bla_{CMY-6}**, bla_{OXA-9}, bla_{TEM-1}, $bla_{SHV-182}$	*aph(3’)-Ia, acc(6’)-Ib, aadA2, rmtC, qac(E), oqxA, oqxB, fosA_3, mph(A), catA1, sul1, dfrA12*
NDM-Kpn-45	**bla_{NDM-1}**, **$bla_{CTX-M-15}$**, bla_{OXA-1}, bla_{OXA-9}, bla_{TEM-1}, bla_{SHV-67}	*aph(3’)-VI, ant(3’’)-Ia, aac(6’)-Ib-cr, acc(6’)-Ib, acc(3)-IIa, qac(E), oqxA, oqxB, fosA_5, ere(A), catB3, catA1, cmlA1, qnrS1, AAR-2, sul1, tet(A)*
NDM-Kpn-46	**bla_{NDM-1}**, **$bla_{CTX-M-15}$**, bla_{OXA-1}, bla_{OXA-9}, bla_{TEM-1}, bla_{SHV-67}	*aph(3’)-VI, ant(3’’)-Ia, aac(6’)-Ib-cr, acc(6’)-Ib, acc(3)-IIa, qac(E), oqxA, oqxB, fosA_5, ere(A), catB3, catA1, cmlA1, qnrS1, AAR-2, sul1, tet(A)*
NDM-Kpn-47	**bla_{NDM-5}**, **bla_{DHA-1}**, bla_{OXA-1}, bla_{TEM-1}, $bla_{SHV-182}$	*aadA2, aph(3’)-Ia, rmtB, aac(6’)-Ib-cr, qacE, oqxA, oqxB, fosA_5, mph(A), catB3, catA1, qnrB4, ARR-3, sul1, dfrA12*
NDM-Kpn-48	**bla_{NDM-1}**, **$bla_{CTX-M-15}$**, bla_{OXA-1}, bla_{OXA-9}, bla_{TEM-1}, bla_{SHV-6}	*aph(3’)-VI, ant(3’’)-Ia, aac(6’)-Ib-cr, acc(6’)-Ib, acc(3)-IIa, qac(E), oqxA, oqxB, fosA_5, ere(A), catB3, catA1, cmlA1, qnrS1, AAR-2, sul1, tet(A)*
NDM-Kpn-49	**bla_{NDM-1}**, **$bla_{CTX-M-15}$**, bla_{OXA-9}, bla_{TEM-1}, bla_{SHV-11}	*aph(3’)-VI, aadA1, aac(6’)-Ib-cr, acc(3)-IIa, qac(E), oqxA, oqxB, fosA_5, qnrS1, AAR-3, sul1*
NDM-Kpn-50	**bla_{NDM-5}**, **bla_{DHA-1}**, bla_{OXA-1}, bla_{TEM-1}, $bla_{SHV-182}$	*aadA2, aph(3’)-Ia, rmtB, aac(6’)-Ib-cr, qacE, oqxA, oqxB, fosA_5, mph(A), catB3, catA1, qnrB4, ARR-3, sul1, dfrA12*
NDM-Kpn-51	**bla_{NDM-1}**, **$bla_{CTX-M-15}$**, bla_{OXA-1}, bla_{OXA-9}, bla_{TEM-1}, bla_{SHV-11}	*aph(3’)-VI, ant(3’’)-Ia, aac(6’)-Ib-cr, acc(6’)-Ib, acc(3)-IIa, qac(E), oqxA, oqxB, fosA_5, ere(A), catB3, catA1, cmlA1, qnrS1, AAR-2, sul1, tet(A)*
NDM-Kpn-52	**bla_{NDM-5}**, **bla_{DHA-1}**, bla_{OXA-1}, bla_{TEM-1}, $bla_{SHV-182}$	*aadA2, aph(3’)-I, rmtB, aac(6’)-Ib-cr, qacE, oqxA, oqxB, fosA_5, mph(A), catB3, catA1, qnrB4, ARR-3, sul1, dfrA12*
NDM-Kpn-53	**bla_{NDM-1}**, **$bla_{CTX-M-15}$**, bla_{OXA-1}, bla_{TEM-1}, bla_{SHV-1}	*aph(3’)-VI, armA, aac(6’)-Ib-cr, qac(E), oqxA, oqxB, fosA_5, msr(E), mph(A), mph(E), catB3, catA1, qnrS1, sul1, sul2, dfrA5*
NDM-Kpn-54	**bla_{NDM-1}**, **$bla_{CTX-M-15}$**, **bla_{CMY-6}**, bla_{TEM-1}, bla_{SHV-28}	*aac(6’)-Ib3, rmtC, qac(E), oqxA, oqxB, fosA_5, sul1*
NDM-Kpn-55	**bla_{NDM-5}**, **$bla_{CTX-M-15}$**, bla_{OXA-1}, bla_{TEM-1}, bla_{SHV-11}	*aadA2, aac(6’)-Ib-cr, rmtB, qacE, oqxA, oqxB, fosA_5, mph(A), erm(B), catB3, qnrS1, ARR-3, sul1, dfrA12*
NDM-Kpn-56	**bla_{NDM-1}**, **bla_{OXA-48}**, **$bla_{CTX-M-1}$**, bla_{SHV-11}	*aph(3’)-VI, aadA2, armA, qac(E), oqxA, oqxB, fosA_5, msr(E), mph(A), mph(E), sul1, tet(A), dfrA12*
NDM-Kpn-57	**bla_{NDM-1}**, **$bla_{CTX-M-15}$**, **bla_{OXA-1}**, bla_{TEM-1}, bla_{SHV-1}	*aph(3’)-VI, aac(6’)-Ib-cr, armA1, qac(E), oqxA1, oqxB1, fosA_3, msr(E), mph(A), mph(E), catB3, catA1, qnrS1, sul1, sul2, dfrA5*
NDM-Kpn-58	**bla_{NDM-1}**, **bla_{CMY-6}**, **$bla_{CTX-M-15}$**, $bla_{SHV-100}$	*aac(6’)-Ib-3, rmtC, qac(E), oqxA, oqxB, fosA_3, sul1*
NDM-Kpn-59	**bla_{NDM-1}**, **$bla_{CTX-M-15}$**, bla_{OXA-1}, bla_{OXA-9}, bla_{TEM-1}, bla_{SHV-11}	*aph(3’)-VI, armA1, qac(E), oqxA1, oqxB1, fosA_5, msr(E), mph(A), mph(E), catB3, qnrS1, ARR-3, sul1, sul2, dfrA5*
NDM-Kpn-60	**bla_{NDM-1}**, **$bla_{CTX-M-15}$**, bla_{OXA-1}, bla_{OXA-9}, bla_{TEM-1}, bla_{SHV-11}	*aph(3’)-VI, aac(6’)-Ib, ant(3’’)-Ia, armA1, aac(6’)-Ib-cr, qac(E), oqxA1, oqxB1, fosA_5, msr(E), mph(A), mph(E), catB3, qnrS1, ARR-3, sul1, sul2, dfrA5*
NDM-Kpn-61	**bla_{NDM-1}**, **$bla_{CTX-M-15}$**, bla_{OXA-1}, bla_{OXA-9}, $bla_{TEM-1}$$bla_{SHV-67}$	*aph(3’)-VI, aac(6’)-Ib, ant(3’’)-Ia, armA1, aac(6’)-Ib-cr, qac(E), oqxA1, oqxB1, fosA_3, fosA_5, msr(E), mph(A), mph(E), catB3, qnrS1, ARR-3, sul1, sul2, dfrA5*
NDM-Kpn-62	**bla_{NDM-1}**, **bla_{KPC-3}**, **$bla_{CTX-M-15}$**, bla_{OXA-1}, $bla_{SHV-182}$	*aac(6’)-Ib-cr, aac(6’)-Ib-1, acc(3)-IIa, oqxA1, oqxB1, fosA_3, catB3, dfrA14*
NDM-Kpn-63	**bla_{NDM-1}**, **$bla_{CTX-M-15}$**, bla_{OXA-1}, bla_{SHV-1}, $bla_{SHV-182}$	*aac(6’)-Ib-1, aac(6’)-Ib-cr, acc(3)-IIa, oqxA1, oqxB1, fosA_3, catB3, dfrA14*
NDM-Kpn-64	**bla_{NDM-1}**, bla_{SHV-27}	*aph(3’)-VI, armA1, qacE, oqxA1, oqxB1, fosA_3, msr(E), mph(A), mph(E), qnrS1, sul1, sul2, dfrA5*
NDM-Kpn-65	**bla_{NDM-1}**, **bla_{OXA-48}**, **$bla_{CTX-M-15}$**, bla_{SHV-67}	*aph(3’)-VI, aadA2, armA, qacE, oqxA1, oqxB1, fosA_5, msr(E), mph(A), mph(E), sul1, tet(A), dfrA12*
NDM-Kpn-66	**bla_{NDM-1}**, **$bla_{CTX-M-15}$**, bla_{OXA-1}, bla_{OXA-9}, bla_{TEM-1}, bla_{SHV-11}	*aph(3’)-VI, aac(6’)-Ib-1, ant(3’’)-Ia, armA1, aac(6’)-Ib-cr, qacE, oqxA1, oqxB1, fosA_5, msr(E), mph(A), mph(E), catB3, qnrS1, ARR-3, sul1, sul2, dfrA5*
NDM-Kpn-67	**bla_{NDM-1}**, **$bla_{CTX-M-15}$**, bla_{OXA-1}, $bla_{OXA-9}$$bla_{TEM-1}$, bla_{SHV-11}	*aph(3’)-VI, aac(6’)-Ib-1, aadA1, armA1, aac(6’)-Ib-cr, qacE, oqxA1, oqxB1, fosA_5, msr(E), mph(A), mph(E), catB3, qnrS1, ARR-3, sul1, sul2, dfrA5*
NDM-Kpn-68	**bla_{NDM-1}**, **$bla_{CTX-M-1}$**, bla_{OXA-1}, bla_{OXA-9}, bla_{TEM-1}, bla_{SHV-11}	*aph(3’)-VI, aac(6’)-Ib-1, ant(3’’)-Ia, armA1, aac(6’)-Ib-cr, qacE, oqxA1, oqxB1, fosA_5, msr(E), mph(A), mph(E), catB3, qnrS1, ARR-3, sul1, sul2, dfrA5*
NDM-Kpn-69	**bla_{NDM-1}**, **$bla_{CTX-M-15}$**, bla_{OXA-1}, bla_{OXA-9}, bla_{TEM-1}, bla_{SHV-11}	*aph(3’)-VI, aac(6’)-Ib-1, ant(3’’)-Ia, aac(6’)-Ib-cr, qacE, oqxA1, oqxB1, fosA_5, msr(E), mph(A), mph(E), catB3, qnrS1, ARR-3, sul1, sul2, dfrA5*

NDM-Kpn-70	***bla***$_{NDM-1}$, ***bla***$_{CTX-M-15}$, *bla*$_{OXA-1}$, *bla*$_{TEM-1A}$, *bla*$_{SHV-12}$	*aac(6')-Ib-1, ant(3'')-Ia, aac(6')-Ib-cr, aac(3')-IIa, oqxA1, oqxB1, fosA_5, catB3, dfrA14*
NDM-Kpn-71	***bla***$_{NDM-1}$, *bla*$_{SHV-27}$, *bla*$_{SHV-1}$	*aph(3')-VI, armA1, qacE, oqxA1, oqxB1, fosA_3, msr(E), mph(A), mph(E), qnrS1, sul1, sul2, tet(D), dfrA5*
NDM-Kpn-72	***bla***$_{NDM-5}$, ***bla***$_{CTX-M-15}$, *bla*$_{OXA-1}$, *bla*$_{TEM-1}$, *bla*$_{SHV-81}$	*aph(6')-Id, aadA2, aac(6')-Ib-cr, rmtB, aph(3')-Ib, qacE, oqxA1, oqxB1, fosA_5, mph(A), erm(B), catB3, qnrB1, sul1, sul2, dfrA12*
NDM-Kpn-73	***bla***$_{NDM-1}$, ***bla***$_{CTX-M-1}$, *bla*$_{OXA-1}$, *bla*$_{SHV-182}$	*aac(6')-Ib-1, aac(6')-Ib-cr, aac(3')-IIa, oqxA1, oqxB1, fosA_5, catB3, dfrA14*
NDM-Kpn-74	***bla***$_{NDM-1}$, ***bla***$_{OXA-48}$, ***bla***$_{CTX-M-15}$, *bla*$_{SHV-67}$	*aph(3')-VI, aadA2, armA1, qacE, oqxA1, oqxB1, fosA_5, msr(E), mph(A), mph(E), sul1, tet(A), dfrA12*
NDM-Kpn-75	***bla***$_{NDM-1}$, ***bla***$_{CTX-M-15}$, *bla*$_{OXA-1}$, *bla*$_{OXA-9}$, *bla*$_{TEM-1}$, *bla*$_{SHV-11}$	*aph(3')-VI, aac (6')-Ib-1, aadA1, aac (6')-Ib-cr, qacE, oqxA1, oqxB1, fosA_5, catB3, qnrS1, ARR-3, sul1*
NDM-Kpn-76	***bla***$_{NDM-1}$, *bla*$_{SHV-27}$, *bla*$_{SHV-1}$	*aph(3')-VI, armA1, qacE, oqxA1, oqxB1, fosA_3, msr(E), mph(A), mph(E), qnrS1, sul1, sul2, tet(D), dfrA5*
NDM-Kpn-77	***bla***$_{NDM-1}$, ***bla***$_{OXA-48}$, ***bla***$_{CTX-M-15}$, *bla*$_{SHV-11}$	*aph(3')-VI, aadA2, armA1, qacE, oqxA1, oqxB1, fosA_5, msr(E), mph(A), mph(E), sul1, tet(A), dfrA12*
NDM-Kpn-78	***bla***$_{NDM-5}$, ***bla***$_{CTX-M-15}$, *bla*$_{SHV-27}$	*aph(6)-Id, aadA2, aph(3')-Ib, qacE, oqxA1, oqxB1, fosA_3, mph(A), catA2, qnrS1, sul1, sul2, dfrA12*
NDM-Kpn-79	***bla***$_{NDM-1}$, *bla*$_{SHV-1}$	*aph(3')-VI, armA1, qacE, oqxA1, oqxB1, fosA_3, msr(E), mph(A), mph(E), qnrS1, sul1, sul2, tet(D), dfrA5*
NDM-Kpn-80	***bla***$_{NDM-1}$, *bla*$_{OXA-10}$, *bla*$_{SHV-33}$	*ant(2'')-Ia, aadA1, armA, qacE, oqxA, oqxB, fosA3, fosA6, msr(E), mph(E), cmlA5, ARR-2, sul1, sul3, dfrA12*
NDM-Kpn-81	***bla***$_{NDM-1}$, ***bla***$_{CTX-M-15}$, *bla*$_{OXA-1}$, *bla*$_{OXA-9}$, *bla*$_{TEM-1A}$, *bla*$_{SHV-11}$,	*aph(3')-VI, aac (6')-Ib-1, ant(3'')-Ia, aac(6')-Ib-cr, aadA2, armA, qacE, oqxA1, oqxB1, fosA_5, msr(E), mph(A), mph(E), qnrS1, catB3, sul1, sul2, dfrA5*
NDM-Kpn-82	***bla***$_{NDM-1}$, ***bla***$_{OXA-48}$, ***bla***$_{CTX-M-15}$, *bla*$_{SHV-11}$	*aph(3')-VI, aadA2, armA, qacE, oqxA1, oqxB1, fosA_5, msr(E), mph(A), mph(E), sul1, tet(A), dfrA12*
NDM-Kpn-83	***bla***$_{NDM-1}$, ***bla***$_{OXA-48}$, ***bla***$_{CTX-M-15}$, *bla*$_{SHV-11}$	*aph(3')-VI, aadA2, armA, qacE, oqxA1, oqxB1, fosA_5, msr(E), mph(A), mph(E), sul1, tet(A), dfrA12*
NDM-Kpn-84	***bla***$_{NDM-1}$, ***bla***$_{CTX-M-15}$, *bla*$_{OXA-1}$, *bla*$_{OXA-9}$, *bla*$_{SHV-11}$	*aac(6')-Ib, aac(6')-Ib-cr, aac(3')-IIa, oqxA1, oqxB1, fosA_3, catB3, dfrA14*
NDM-Kpn-85	***bla***$_{NDM-1}$, ***bla***$_{OXA-48}$, ***bla***$_{CTX-M-15}$, *bla*$_{OXA-9}$, *bla*$_{SHV-11}$	*aph(3')-VI, aadA2, armA, qacE, oqxA1, oqxB1, fosA_5, msr(E), mph(A), mph(E), sul1, tet(A), dfrA12*
NDM-Kpn-86	***bla***$_{NDM-1}$, ***bla***$_{CTX-M-15}$, *bla*$_{OXA-1}$, *bla*$_{OXA-9}$, *bla*$_{TEM-1A}$, *bla*$_{SHV-11}$,	*aph(3')-VI, aac (6')-Ib-1, ant(3'')-Ia, aac(6')-Ib-cr, armA, qacE, oqxA1, oqxB1, fosA_5, msr(E), mph(A), mph(E), qnrS1, catB3, ARR-3, sul1, sul2, dfrA5*
NDM-Kpn-87	***bla***$_{NDM-1}$, ***bla***$_{VIM-1}$, ***bla***$_{CTX-M-15}$, *bla*$_{TEM-1A}$, *bla*$_{SHV-11}$	*aph(3')-VI, aac (6')-Ib-1, ant(3'')-Ia, aac(6')-Ib-cr, qacE, oqxA1, oqxB1, fosA_5, mph(A), qnrS1, catB2, ARR-3, sul1, dfrA12*
NDM-Kpn-88	***bla***$_{NDM-1}$, ***bla***$_{VIM-1}$, ***bla***$_{CTX-M-15}$, *bla*$_{OXA-9}$, *bla*$_{TEM-1A}$, *bla*$_{SHV-12}$	*aph(3')-VI, aph(3')-XV, aac (6')-Ib-1, ant(3'')-Ia, aac(6')-Ib-cr, qacE, oqxA1, oqxB1, fosA_5, mph(A), qnrS1, catB2, ARR-3, sul1, dfrA14*
NDM-Kpn-89	***bla***$_{NDM-1}$, *bla*$_{TEM-1A}$, *bla*$_{SHV-11}$	*ant(2'')-Ia, aac(6')-Ib, aph(3')-Ia, oqxA1, oqxB1, fosA_3, qnrB19, dfrA14*
NDM-Kpn-90	***bla***$_{NDM-1}$, ***bla***$_{VIM-1}$, ***bla***$_{CTX-M-15}$, *bla*$_{OXA-9}$, *bla*$_{TEM-1A}$, *bla*$_{SHV-12}$	*aph(3')-VI, aph(3')-XV, aac (6')-Ib-1, ant(3'')-Ia, aac(6')-Ib-cr, qacE, oqxA1, oqxB1, fosA_5, mph(A), qnrS1, catB2, ARR-3, sul1, dfrA14*
NDM-Kpn-91	***bla***$_{NDM-1}$, ***bla***$_{OXA-48}$, ***bla***$_{CTX-M-15}$, *bla*$_{SHV-67}$	*aph(3')-VI, aadA2, armA, qacE, oqxA1, oqxB1, fosA_5, msr(E), mph(A), mph(E), sul1, tet(A), dfrA12*
NDM-Kpn-92	***bla***$_{NDM-1}$, *bla*$_{SHV-27}$	*aph(3')-VI, armA, qacE, oqxA1, oqxB1, fosA_5, msr(E), mph(A), mph(E), sul1, sul2, tet(D), dfrA5*
NDM-Kpn-93	***bla***$_{NDM-1}$, ***bla***$_{VIM-1}$, ***bla***$_{CTX-M-15}$, *bla*$_{OXA-9}$, *bla*$_{TEM-1A}$, *bla*$_{SHV-11}$	*aph(3')-XV, aac (6')-Ib-1, ant(3'')-Ia, qacE, oqxA1, oqxB1, fosA_5, mph(A), qnrS1, catB2, sul1, dfrA14*
NDM-Kpn-94	***bla***$_{NDM-1}$, ***bla***$_{CTX-M-15}$, *bla*$_{OXA-1}$, *bla*$_{TEM-1A}$, *bla*$_{SHV-11}$	*aph(3')-VI, armA, aac(6')-Ib-cr , qacE, oqxA1, oqxB1, fosA_5, msr(E), mph(A), mph(E), qnrS1, catB3, ARR-3, sul1, sul2, dfrA5*
NDM-Kpn-95	***bla***$_{NDM-1}$, *bla*$_{SHV-27}$	*aph(3')-VI, armA, qacE, oqxA1, oqxB1, fosA_3, msr(E), mph(A), mph(E), sul1, sul2, tet(D), dfrA5*
NDM-Kpn-96	***bla***$_{NDM-1}$, ***bla***$_{OXA-48}$, ***bla***$_{CTX-M-15}$, *bla*$_{SHV-11}$	*aph(3')-VI, aadA2, armA, qacE, oqxA1, oqxB1, fosA_5, msr(E), mph(A), mph(E), sul1, tet(A), dfrA12*
NDM-Kpn-97	***bla***$_{NDM-1}$, ***bla***$_{CTX-M-15}$, ***bla***$_{CMY-6}$, *bla*$_{OXA-181}$, *bla*$_{TEM-1B}$, *bla*$_{SHV-28}$	*aac(6')-Ib3, ant(3'')-Ia, aph(3')-Ia, aph(3')-Ib, rmtC, qac(E), oqxA, oqxB, fosA_5, erm(B), mph(A), catA2, qnrS1, sul1, sul2, tet(A), dfrA12*
NDM-Kpn-98	***bla***$_{NDM-1}$, ***bla***$_{CTX-M-15}$, *bla*$_{OXA-1}$, *bla*$_{TEM-1A}$, *bla*$_{SHV-12}$	*ant(2'')-Ia, aac(6')-Ib, aph(3')-Ia, aac(6')-Ib-cr, oqxA1, oqxB1, fosA_5, catB3, qnrB19, dfrA14*
NDM-Kpn-99	***bla***$_{NDM-1}$, ***bla***$_{CTX-M-15}$, *bla*$_{OXA-1}$, *bla*$_{SHV-182}$	*aac(6')-Ib, aac(6')-Ib-cr, oqxA1, oqxB1, fosA_3, catB3, dfrA14*
NDM-Kpn-100	***bla***$_{NDM-5}$, *bla*$_{SHV-1}$	*aadA2, qacE, oqxA, oqxB, fosA_6, sul1, dfrA12*
NDM-Kpn-101	***bla***$_{NDM-1}$, ***bla***$_{CTX-M-15}$, *bla*$_{OXA-1}$, *bla*$_{TEM-1B}$, *bla*$_{SHV-182}$	*aac(6')-Ib-cr, rmtC, qacE, oqxA1, oqxB1, fosA_3, catB3, qnrS1, tet(A), dfrA1*
NDM-Kpn-102	***bla***$_{NDM-1}$, ***bla***$_{VIM-1}$, ***bla***$_{CTX-M-15}$, *bla*$_{OXA-9}$, *bla*$_{TEM-1A}$, *bla*$_{SHV-12}$	*aph(3')-VI, aac (6')-Ib-1, ant(3'')-Ia, aac(6')-Ib-cr, qacE, oqxA1, oqxB1, fosA_5, mph(A), qnrS1, catB2, ARR-3, sul1, dfrA14*
NDM-Kpn-103	***bla***$_{NDM-1}$, ***bla***$_{OXA-48}$, ***bla***$_{CTX-M-15}$, *bla*$_{SHV-11}$	*aph(3')-VI, aadA2, armA, qacE, oqxA1, oqxB1, fosA_5, msr(E), mph(A), mph(E), sul1, tet(A), dfrA12*
NDM-Kpn-104	***bla***$_{NDM-1}$, ***bla***$_{CTX-M-15}$, *bla*$_{OXA-1}$, *bla*$_{SHV-182}$	*aac(6')-Ib, aac(6')-Ib-cr, oqxA1, oqxB1, fosA_3, catB3, dfrA14*
NDM-Kpn-105	***bla***$_{NDM-1}$, *bla*$_{SHV-27}$	*aph(3')-VI, armA, msr(E), mph(A), mph(E), qnrS1, sul1, sul2, tet(D), dfrA5*
NDM-Kpn-106	***bla***$_{NDM-1}$, ***bla***$_{CTX-M-15}$, *bla*$_{OXA-1}$, *bla*$_{SHV-1}$	*aac(6')-Ib, aac(6')-Ib-cr, aac(3')-IIa, oqxA1, oqxB1, fosA_3, catB3, dfrA14*
NDM-Kpn-107	***bla***$_{NDM-5}$, ***bla***$_{OXA-48}$, ***bla***$_{CTX-M-15}$, ***bla***$_{CTX-M-14b}$, *bla*$_{OXA-9}$, *bla*$_{TEM-1C}$, *bla*$_{SHV-1}$	*aac(6')-Ib, aph(3')-Ia, aph(3')-Ib, aph(3')-VI, aph(6')-Id, aadA1, oqxA1, oqxB1, fosA_3, catB3, mph(A), catA1, qnrS1, sul2, tet(A)*

NDM-Kpn-108	***bla***$_{\textbf{NDM-1}}$, ***bla***$_{\textbf{CTX-M-15}}$, *bla*$_{\text{OXA-1}}$, *bla*$_{\text{TEM-1A}}$, *bla*$_{\text{SHV-12}}$	*ant(2’’)-Ia, aac(6’)-Ib, aph(3’)-Ia, aac(6’)-Ib-cr, aac(3’)-IIa, oqxA1, oqxB1, fosA_3, catB3, qnrB19, dfrA14*
NDM-Kpn-109	***bla***$_{\textbf{NDM-1}}$, ***bla***$_{\textbf{CTX-M-15}}$, *bla*$_{\text{OXA-1}}$, *bla*$_{\text{SHV-11}}$	*aac(6’)-Ib, aac(6’)-Ib-cr, oqxA1, oqxB1, fosA_3, catB3, dfrA14*
NDM-Kpn-110	***bla***$_{\textbf{NDM-1}}$, ***bla***$_{\textbf{CTX-M-15}}$, *bla*$_{\text{OXA-1}}$, *bla*$_{\text{SHV-11}}$	*aac(6’)-Ib, aac(6’)-Ib-cr, oqxA1, oqxB1, fosA_3, catB3, dfrA14*
NDM-Kpn-111	***bla***$_{\textbf{NDM-5}}$, ***bla***$_{\textbf{CTX-M-15}}$, *bla*$_{\text{OXA-1}}$, *bla*$_{\text{OXA-9}}$, *bla*$_{\text{TEM-1A}}$, *bla*$_{\text{SHV-11}}$	*aac(6’)-Ib, aph(3’)-VI, aadA1, aac(6’)-Ib-cr, qacE, oqxA1, oqxB1, fosA_5, catB3, qnrS1, ARR-3, sul1*
NDM-Kpn-112	***bla***$_{\textbf{NDM-5}}$, ***bla***$_{\textbf{CMY-2}}$, ***bla***$_{\textbf{CTX-M-15}}$, *bla*$_{\text{OXA-9}}$, *bla*$_{\text{TEM-1A}}$, *bla*$_{\text{SHV-11}}$	*aph(6’)-Id, aac(6’)-Ib, aac(3’)-II, aph(3’)-VI, aph(3’’)-Ib, aadA1, qacE, oqxA1, oqxB1, fosA_5, mph(A), floR, catA1, qnrS1, sul1, sul2, tet(A), dfrA5*
NDM-Kpn-113	***bla***$_{\textbf{NDM-1}}$, ***bla***$_{\textbf{OXA-48}}$, ***bla***$_{\textbf{CTX-M-15}}$, *bla*$_{\text{SHV-11}}$	*aph(3’)-VI, aadA1, armA, qacE, oqxA1, fosA_5, msrE, mph(A), mph(E), sul1, tet(A), dfrA12*
NDM-Kpn-114	***bla***$_{\textbf{NDM-1}}$, ***bla***$_{\textbf{CTX-M-15}}$, *bla*$_{\text{OXA-1}}$, *bla*$_{\text{SHV-1}}$	*aac(6’)-Ib, aac(6’)-Ib-cr, oqxA1, oqxB1, fosA_3, catB3, dfrA14*
NDM-Kpn-115	***bla***$_{\textbf{NDM-1}}$, ***bla***$_{\textbf{CTX-M-15}}$, *bla*$_{\text{OXA-1}}$, *bla*$_{\text{TEM-1A}}$, *bla*$_{\text{SHV-12}}$	*ant(2’’)-Ia, aac(6’)-Ib, aph(3’)-Ia, aac(6’)-Ib-cr, aac(3’)-IIa, oqxA1, oqxB1, fosA_3, catB3, qnrB19, dfrA14*
NDM-Kpn-116	***bla***$_{\textbf{NDM-1}}$, ***bla***$_{\textbf{VIM-1}}$, ***bla***$_{\textbf{CTX-M-15}}$, *bla*$_{\text{OXA-9}}$, *bla*$_{\text{TEM-1A}}$, *bla*$_{\text{SHV-12}}$	*aph(3’)-VI, aac (6’)-Ib-1, ant(3’’)-Ia, aac(6’)-Ib-cr, qacE, oqxA1, oqxB1, fosA_5, mph(A), qnrS1, catB2, ARR-3, sul1, dfrA14*
NDM-Kpn-117	***bla***$_{\textbf{NDM-5}}$, *bla*$_{\text{OXA-10}}$, *bla*$_{\text{SHV-1}}$	*aac(6’)-II, aph(3’)-VI, qacE, oqxA1, oqxB1, fosA_3, qnrS1, sul1*
NDM-Kpn-118	***bla***$_{\textbf{NDM-1}}$, ***bla***$_{\textbf{CTX-M-15}}$, *bla*$_{\text{OXA-1}}$, *bla*$_{\text{SHV-182}}$	*aac(6’)-Ib, aac(6’)-Ib-cr, oqxA1, oqxB1, fosA_3, catB3, dfrA14*
NDM-Kpn-119	***bla***$_{\textbf{NDM-1}}$, ***bla***$_{\textbf{CTX-M-15}}$, *bla*$_{\text{OXA-1}}$, *bla*$_{\text{SHV-11}}$	*aph(3’)-VI, aph(3)-Ia, armA, aac(6’)-Ib-cr, qacE, oqxA1, oqxB1, fosA_5, mph(A), mph(E), qnrS1, catB3, ARR-3, sul1, sul2, dfrA14*
NDM-Kpn-120	***bla***$_{\textbf{NDM-1}}$, ***bla***$_{\textbf{CTX-M-15}}$, *bla*$_{\text{OXA-1}}$, *bla*$_{\text{OXA-9}}$, *bla*$_{\text{TEM-1A}}$, *bla*$_{\text{SHV-11}}$	*aph(3’)-VI, aac(6’)-Ib, ant(3’’)-Ia, armA, aac(6’)-Ib-cr, qacE, oqxA1, oqxB1, fosA_5, msrE, mph(A), mph(E), catB3, qnrS1, ARR-3, sul1, sul2, dfrA5*
NDM-Kpn-121	***bla***$_{\textbf{NDM-1}}$, ***bla***$_{\textbf{OXA-48}}$, ***bla***$_{\textbf{CTX-M-15}}$, *bla*$_{\text{SHV-67}}$	*aph(3’)-VI, aadA2, armA, qacE, oqxA1, oqxB1, fosA_5, msr(E), mph(A), mph(E), sul1, tet(A), dfrA12*
NDM-Kpn-122	***bla***$_{\textbf{NDM-1}}$, ***bla***$_{\textbf{CTX-M-15}}$, *bla*$_{\text{OXA-1}}$, *bla*$_{\text{TEM-1B}}$, *bla*$_{\text{SHV-182}}$	*aph(6’)-Id, aph(3’)-VI, aph(3’)-VIa, armA, aac(6’)-Ib-cr , qacE, oqxA1, oqxB1, fosA_3, msr(E), mph(A), mph(E), qnrS1, catB3, catA1, sul1, sul2, tet(A), dfrA5, dfrA1*
NDM-Kpn-123	***bla***$_{\textbf{NDM-1}}$, ***bla***$_{\textbf{VIM-1}}$, ***bla***$_{\textbf{CTX-M-15}}$, *bla*$_{\text{OXA-9}}$, *bla*$_{\text{TEM-1A}}$, *bla*$_{\text{SHV-12}}$	*aph(3’)-VI, aac (6’)-Ib-1, ant(3’’)-Ia, aac(6’)-Ib-cr, qacE, oqxA1, oqxB1, fosA_5, mph(A), qnrS1, catB2, ARR-3, sul1, dfrA14*
NDM-Kpn-124	***bla***$_{\textbf{NDM-5}}$, ***bla***$_{\textbf{OXA-48}}$, ***bla***$_{\textbf{CTX-M-15}}$, *bla*$_{\text{OXA-9}}$, *bla*$_{\text{TEM-1C}}$, *bla*$_{\text{SHV-1}}$	*aac(6’)-Ib, aph(3’)-Ia, aph(3’)-Ib, aph(3’)-VI, aph(6’)-Id, aadA1, armA, qacE, oqxA1, oqxB1, fosA_3, catB3, msr(E), mph(A), mph(E), catA1, qnrS1, sul1, sul2, tet(A), dfrA5*
NDM-Kpn-125	***bla***$_{\textbf{NDM-1}}$, ***bla***$_{\textbf{CTX-M-15}}$, *bla*$_{\text{OXA-1}}$, *bla*$_{\text{OXA-9}}$, *bla*$_{\text{TEM-1A}}$, *bla*$_{\text{SHV-1}}$	*aac(6’)-Ib, aac(6’)-Ib-cr, oqxA1, oqxB1, fosA_3, catB3, dfrA14*
NDM-Kpn-126	***bla***$_{\textbf{NDM-1}}$, ***bla***$_{\textbf{OXA-48}}$, ***bla***$_{\textbf{CTX-M-15}}$, *bla*$_{\text{SHV-11}}$	*aph(3’)-VI, aadA2, armA, qacE, oqxA1, fosA_5, msr(E), mph(A), mph(E), sul1, tet(A), dfrA12*
