# Supplementary material for: Molecular Characterization of Multidrug-Resistant and Hypervirulent New Delhi Metallo-Beta-Lactamase Klebsiella pneumoniae in Lazio, Italy: A Five-Year Retrospective Study
Source: Antibiotics (Basel). 2024 Nov 5;13(11):1045. doi: 10.3390/antibiotics13111045 (PMC11590914; doi:10.3390/antibiotics13111045)
Supplement: Supplementary file 1 [file antibiotics-13-01045-s001.zip › Supplementary Table S3.pdf]

Supplementary Table S3. Antimicrobial susceptibility results for NDM-Kpn clinical isolates vs Resistance genes and STs

Strain	Date	ST[1]	Antibiotic MIC values (mg/L)[2]										Resistance genes
			CZA	ETP	IPM	MEM	GEN	AMK	CIP	TZP	SXT	COL	
**NDM-Kpn-1**	22/05/19	11	>256, R	>1, R	>8, R	>8, R	2, S	≤4, S	>1, R	>16/4, R	≤1/19, S	0.125, S	*aac(6)-Ib, fosA_3, oqxA, oqxB, dfrA14*
**NDM-Kpn-2**	12/07/19	11	>256, R	>1, R	>8, R	>8, R	≤1, S	32, R	>1, R	>16/4, R	>4/76, R	0.5, S	*fosA_3, oqxA, oqxB, dfrA14*
**NDM-Kpn-3**	18/07/19	15	>256, R	>1, R	>8, R	>8, R	>16, R	32, R	>=4, R	>16/4, R	>4/76, R	0.5, S	*aac(6)-Ib, rmtC, fosA_3, oqxA, oqxB, sul1, sul2, dfrA14*
**NDM-Kpn-4**	29/07/19	147	>256, R	>1, R	>8, R	>8, R	>16, R	32, R	>=4, R	>16/4, R	>4/76, R	16, R	*aph(3')-VI, aac(6')-Ib, aadA1, armA, aac(6')-Ib-cr, fosA_3, fosA_5, msr(E), mph(A), mph(E), catB3, qnrS1, oqxA, oqxB, ARR-3, sul1, sul2, dfrA5*
**NDM-Kpn-5**	29/08/19	383	>256, R	>1, R	>8, R	>8, R	2, S	32, R	>=4, R	>16/4, R	>4/76, R	0.5, S	*aph(3')-VI, aac(6')-Ib, aadA1, aph(3')-Ia, armA, fosA_3, fosA_5, msr(E), mph(A), mph(E), catA1, qnrS1, oqxA, oqxB, sul1, sul2, tet(A), dfrA5*
**NDM-Kpn-6**	13/09/19	15	>256, R	>1, R	>8, R	>8, R	>16, R	32, R	>=4, R	>16/4, R	>4/76, R	2, S	*aac(6')-Ib, rmtC ,fosA_3, oqxA, oqxB, sul1, sul2, dfrA14*
**NDM-Kpn-7**	27/09/19	307	>256, R	>1, R	>8, R	>8, R	>4, R	8, S	>1, R	>16/4, R	>4/76, R	0.5, S	*aph(6)-Id, aac(6')-II, aph(3')-VI, aac(3')-II, aac(6')-Ib-cr, aph(3'')-Ib, fosA_3, catB3, qnrB1, qnrS1, oqxA, oqxB, sul1, sul2, tet(A), dfrA14*
**NDM-Kpn-8**	14/11/19	15	>256, R	>1, R	>8, R	>8, R	>16, R	32, R	>=4, R	>16/4, R	>4/76, R	16, R	*aac(6')-Ib, rmtC, fosA_3, oqxA, oqxB, sul1, sul2*
**NDM-Kpn-9**	18/11/19	4853	>256, R	>1, R	>8, R	>8, R	>16, R	32, R	>=4, R	>16/4, R	>4/76, R	0,5, S	*aph(6)-Id, aac(3')-IIa, aac(6')-Ib, aph(3')-VI, aph(3'')-Ib, rmtC, mph(A), fosA_3, catA1, oqxA, oqxB, sul1, tet(A)*
**NDM-Kpn-10**	18/11/19	383	>256, R	>1, R	>8, R	>8, R	>16, R	32, R	>=4, R	>16/4, R	>4/76, R	0,5, S	*aph(6)-Id, aac(6')-Ib, aph(3')-VI, rmtC, mph(A), aac(6')-Ib-cr, aph(3'')-Ib, fosA_3, catA1, qnrS1, oqxA, oqxB, sul1, sul2, tet(A)*
**NDM-Kpn-11**	30/12/19	17	>256, R	>1, R	>8, R	>8, R	>16, R	4, S	>=4, R	>16/4, R	>4/76, R	0.5, S	*aph(6)-Id, aac(3')-IIa, addA1, aac(6')-Ib-cr, fosA_3, catB3, qnrB1, oqxA, oqxB, sul1, sul2, tet(A), dfrA15*
**NDM-Kpn-12**	06/02/20	147	>256, R	>1, R	>8, R	>8, R	>16, R	32, R	>=4, R	>16/4, R	>4/76, R	0.5, S	*aac(6')-Ib, aph(3')-VI, aph(3')-Ia aac(6')-Ib-cr, aadA1, armA, msr(E), mph(A), mph(E), fosA_5, catB3, qnrS1, oqxA, oqxB, ARR-3, sul1, sul2, dfrA5*
**NDM-Kpn-13**	17/02/20	383	>256, R	>1, R	>8, R	>8, R	>4, R	16, R	>1, R	>16/4, R	≤1/19, S	0.5, S	*aph(6)-Id, aph(3'')-Ib, rmtF, mph(A), fosA_3, fosA_5, catA1, qnrB1, oqxA, oqxB, AAR-2, tet(A)*
**NDM-Kpn-14**	22/04/20	147	>256, R	>1, R	>8, R	>8, R	≤1, S	≤4, S	>1, R	>16/4, R	>4/76, R	0.25, S	*aadA1, aac(6')-Ib-cr, fosA_3, fosA_5, catB3, qnrB1, oqxA, oqxB, sul1, dfrA15*
**NDM-Kpn-15**	23/04/20	383	>256, R	>1, R	>8, R	>8, R	>4, R	>16, R	>1, R	>16/4, R	≤1/19, S	4, R	*aph(6)-Id, aph(3'')-Ib, rmtF, mph(A), fosA_3, fosA_5, catA1, qnrB1, oqxA, oqxB, AAR-2, tet(A)*
**NDM -Kpn-16**	05/05/20	147	>256, R	>1, R	>8, R	>8, R	≤1, S	≤4, S	>1, R	>16/4, R	>4/76, R	0.25, S	*aadA1, aac(6')-Ib-cr, fosA_3, fosA_5, catB3, qnrB1, oqxA, oqxB, sul1, dfrA1*
**NDM -Kpn-17**	30/06/20	29	>256, R	>1, R	>8, R	>8, R	>16, R	8, S	>=4, R	>16/4, R	>4/76, R	0.5, S	*aac(6')-II, aph(3')-VI, acc(3)-II, aadA16, aac(6')-Ib-cr, fosA_3, floR_2, qnrB6, qnrS1, oqxA, oqxB, ARR-3, sul1, sul2, tet(A), tet(D), dfrA27*
**NDM -Kpn-18**	06/10/20	383	>256, R	>1, R	>8, R	>8, R	>4, R	>16, R	>1, R	>16/4, R	≤1/19, S	8, R	*aac(6')-Ib, aph(3')-Ib, rmtF, strA, strB, mph(A), oqxA, oqxB, fosA_3, fosA_5, catA1, qnrB1, AAR-2, tet(A)*
**NDM -Kpn-19**	11/02/21	395	>256, R	>1, R	>8, R	>8, R	>4, R	>16, R	>1, R	>16/4, R	>4/76, R	0.5, S	*aph(3')-VI, armA, aac(6')-Ib-cr, qacE, oqxA, oqxB, fosA_3, msr(E), mph(A), mph(E), catB3, catA1, qnrS1, sul1, sul2, tet(A), dfrA5, dfrA1*

**NDM -Kpn-20**	19/02/21	395	>256, R	>2, R	>8, R	>8, R	>8, R	>16, R	>1, R	>16/4, R	>4/76, R	0.5, S	*aph(3')-VI, armA, aac(6')-Ib-cr, qacE, oqxA, oqxB, fosA_3, msr(E), mph(A), mph(E), catB3, catA1, qnrS1, sul1, sul2, tet(A), dfrA5, dfrA1*
**NDM -Kpn-21**	03/03/21	147	>256, R	>1, R	>8, R	>8, R	>4, R	>16, R	>1, R	>16/4, R	>4/76, R	0.25, S	*aph(3')-VI, acc(6')-Ib, ant(3'')-Ia, aph(3')-Ia, armA, aac(6')-Ib-cr, qac(E), oqxA, oqxB, fosA_5, msr(E), mph(E), mph(A), catB3, qnrB1, sul1, sul2, ARR-3, dfrA5*
**NDM -Kpn-22**	08/03/21	11	>256, R	>1, R	>8, R	>8, R	>4, R	>16, R	>1, R	>64/4, R	>4/76, R	2, R	*acc(6')-Ib, aac(6')-Ib-cr, aac(3)-IIa, oqxA, oqxB, fosA_3, catB3, dfrA14*
**NDM -Kpn-23**	23/03/21	147	>256, R	>1, R	>8, R	>8, R	>4, R	>16, R	>1, R	>16/4, R	>4/76, R	4, R	*ant(3'')-Ia, armA, rmtB, qacE, oqxA, oqxB, fosA_5, erm(B), msr(E), mph(A), mph(E), ere(A), cmlA1, qnrB1, ARR-2, sul1, dfrA12*
**NDM -Kpn-24**	01/04/21	395	>256, R	>1, R	>8, R	>8, R	>4, R	>16, R	>1, R	>16/4, R	>4/76, R	2, S	*aph(3')-VI, armA, aac(6')-Ib-cr, qac(E), oqxA, oqxB, fosA_3, msr(E), mph(A), mph(E), catB3, catA1, qnrS1, sul1, sul2, tet(A), dfrA5, dfrA1*
**NDM -Kpn-25**	01/04/21	147	>256, R	>1, R	>8, R	>8, R	>4, R	>16, R	>1, R	>16/4, R	>4/76, R	4, R	*ant(3'')-Ia, aadA2, armA, rmtB, qacE, oqxA, oqxB, fosA_5, erm(B), msr(E), mph(A), mph(E), ere(A), cmlA1, qnrB1, ARR-2, sul1, dfrA12*
**NDM -Kpn-26**	08/04/21	147	>256, R	>1, R	>8, R	>8, R	≤ 1, S	≤ 4, S	>1, R	>16/4, R	≤ 1/19, S	16, R	*ant(3'')-Ia, aadA2, armA, rmtB, qacE, oqxA, oqxB, fosA_5, erm(B), msr(E), mph(A), mph(E), ere(A), cmlA1, qnrB1, ARR-2, sul1, dfrA12*
**NDM -Kpn-27**	30/04/21	147	>256, R	>1, R	>8, R	>8, R	>4, R	>16, R	>1, R	>16/4, R	>4/76, R	16, R	*ant(3'')-Ia, aadA2, armA, rmtB, qacE, oqxA, oqxB, fosA_5, erm(B), msr(E), mph(A), mph(E), ere(A), cmlA1, qnrB1, ARR-2, sul1, dfrA12*
**NMD-Kpn-28**	15/06/21	11	>256, R	>1, R	>8, R	>8, R	>4, R	>16, R	>1, R	>16/4, R	>4/76, R	2, R	*aac(6')-Ib, aac(6')-Ib-cr, aadA2, aac(3')-IIa, qac(E), oqxA, oqxB, fosA_3, mph(A), catB3, sul1, dfrA12, dfrA14*
**NDM -Kpn-29**	17/06/21	147	>256, R	>1, R	>8, R	>8, R	>4, R	>16, R	>1, R	>16/4, R	>4/76, R	16, R	*ant(3'')-Ia, aadA2, armA, rmtB, qacE, oqxA, oqxB, fosA_5, erm(B), msr(E), mph(A), mph(E), ere(A), cmlA1, qnrB1, ARR-2, sul1, dfrA12*
**NDM -Kpn-30**	17/09/21	395	>256, R	>2, R	>8, R	>8, R	>8, R	>16, R	>1, R	>64/4, R	>4/76, R	0.5, S	*aph(3')-VI, armA, aac(6')-Ib-cr, qac(E), oqxA, oqxB, fosA_3, msr(E), mph(A), mph(E), catB3, catA1, qnrS1, sul1, sul2, tet(A), dfrA5, dfrA1*
**NDM -Kpn-31**	23/11/21	147	>256, R	>2, R	>8, R	>8, R	≤ 1, S	>16, R	>1, R	>64/4, R	>4/76, R	0.5, S	*aph(6)-Id, aph(3')-VI, acc(2')-Ib, acc(6')-Ib, aadA1, aph(3')-Ia, armA, aac(6')-Ib-cr, qac(E), oqxA, oqxB, fosA_3, fosA_5, msr(E), mph(A), mph(E) erm(37), catB3, qnrB1, sul1, sul2, ARR-3, dfrA5*
**NDM-Kpn-32**	10/03/22	147	>256, R	>2, R	>8, R	>8, R	≤ 1, S	>16, R	>1, R	>64/4, R	>4/76, R	0.5, S	*aph(3')-VI, aph(3')-Ia, armA, oqxA, oqxB, fosA_3, fosA_5, msr(E), mph(A), mph(E), qnrS1, sul2,*
**NDM-Kpn-33**	31/03/22	512	>256, R	>1, R	>8, R	>8, R	>4, R	>16, R	>1, R	>16/4, R	>4/76, R	1, S	*aph(3')-Ia, acc(6')-Ib, aadA2, rmtC, qac(E), oqxA, oqxB, fosA_3, msr(E), mph(A), mph(E), catA1, qnrB1, sul1, dfrA12*
**NDM-Kpn-34**	28/03/22	512	>256, R	>1, R	>8, R	>8, R	>4, R	>16, R	>1, R	>16/4, R	>4/76, R	1, S	*aph(3')-Ia, acc(6')-Ib3, aadA2, rmtC, qac(E), oqxA, oqxB, fosA_3, msr(E), mph(A), catA1, sul1, dfrA12*
**NDM-Kpn-35**	15/04/22	395	>256, R	>1, R	>8, R	>8, R	>4, R	>16, R	>1, R	>16/4, R	>4/76, R	1, S	*aph(3')-VI, armA, aac(6')-Ib-cr, qac(E), oqxA, oqxB, fosA_3, msr(E), mph(A), mph(E), catB3, catA1, qnrS1, sul1, sul2, dfrA5*
**NDM-Kpn-36**	18/05/22	11	>256, R	>1, R	>8, R	>8, R	>16, R	>16, R	>=4, R	>64/4, R	≤1/19, S	1, S	*aac(6')-Ib, aac(6')-Ib-cr, aac(3')-IIa, qac(E), oqxA, oqxB, fosA_3, catB3, dfrA14*
**NDM-Kpn-37**	24/05/22	11	>256, R	>1, R	>8, R	>8, R	2, S	16, R	>1, R	>16/4, R	>4/76, R	1, S	*aac(6')-Ib, aac(6')-Ib-cr, aac(3')-Ia, qac(E), oqxA, oqxB, fosA_3, catB3, dfrA14*
**NDM-Kpn-38**	13/06/22	512	>256, R	>1, R	>8, R	>8, R	>4, R	>16, R	>1, R	>16/4, R	>4/76, R	0.5, S	*aph(3')-Ia, acc(6')-Ib3, aadA2, rmtC, qac(E), oqxA, oqxB, fosA_3, mph(A), catA1, sul1, dfrA12*
**NDM-Kpn-39**	30/06/22	11	>256, R	>2, R	8, R	>8, R	>8, R	>16, R	>1, R	>64/4, R	>4/76, R	0.5, S	*aac(3')-IIa, aac(6')-Ib-cr, aadA2, aph(3')-Ia, rmtF, qac(E), oqxA, oqxB, fosA_3, mph(A), catB3, qnrB1, ARR-2, sul1, dfrA12*

**NDM-Kpn-40**	25/07/22	23	>256, R	>1, R	>8, R	>8, R	>4, R	16, R	>1, R	>16/4, R	>4/76, R	1, S	*aph(3')-VI, qac(E), oqxA, oqxB, fosA_3, mph(A), catA1, sul1, sul2, qnrS1, dfrA5, tet(A)*
**NDM-Kpn-41**	26/07/22	147	>256, R	>1, R	>8, R	>8, R	>8, R	>16, R	>1, R	>64/4, R	>4/76, R	0.5, S	*aadA1, aph(3')-VI, armA, aac(6')-Ib-cr, qac(E), oqxA, oqxB, fosA_5, msr(E), mph(A), mph(E), catB3, qnrS1, AAR-3, sul1, sul2, dfrA5*
**NDM-Kpn-42**	05/08/22	11	>256, R	>1, R	>8, R	>8, R	>4, R	16, R	>1, R	>16/4, R	>4/76, R	1, S	*aac(3')-IIa, aac(6')-Ib-cr, aac(6')-Ib, oqxA, oqxB, fosA_3, catB3, dfrA14*
**NDM-Kpn-43**	09/08/22	11	>256, R	>1, R	>8, R	>8, R	>4, R	8, S	>1, R	>16/4, R	≤ 1/19, S	0.5, S	*ant(2'')-Ia, aac(6')-Ib, aac(6')-Ib-cr, aph(3')-Ia, oqxA, oqxB, fosA_3, catB3, qnrB19, dfrA14*
**NDM-Kpn-44**	15/08/22	512	>256, R	>1, R	>8, R	>8, R	>4, R	>16, R	>1, R	>16/4, R	>4/76, R	16, R	*aph(3')-Ia, acc(6')-Ib, aadA2, rmtC, qac(E), oqxA, oqxB, fosA_3, mph(A), catA1, sul1, dfrA12*
**NDM-Kpn-45**	22/08/22	147	>256, R	>2, R	>8, R	>8, R	>8, R	8, S	>1, R	>64/4, R	≤ 1/19, S	0.5, S	*aph(3')-VI, ant(3'')-Ia, aac(6')-Ib-cr, acc(6')-Ib, acc(3)-IIa, qac(E), oqxA, oqxB, fosA_5, ere(A), catB3, catA1, cmlA1, qnrS1, AAR-2, sul1, tet(A)*
**NDM-Kpn-46**	22/08/22	147	>256, R	>1, R	>8, R	>8, R	32, R	8, S	>8, R	>64/4, R	≤ 1/19, S	1, S	*aph(3')-VI, ant(3'')-Ia, aac(6')-Ib-cr, acc(6')-Ib, acc(3)-IIa, qac(E), oqxA, oqxB, fosA_5, ere(A), catB3, catA1, cmlA1, qnrS1, AAR-2, sul1, tet(A)*
**NDM-Kpn-47**	22/08/22	11	>256, R	>2, R	>8, R	>8, R	>8, R	>16, R	>1, R	>64/4, R	>4/76, R	0.5, S	*aadA2, aph(3')-Ia, rmtB, aac(6')-Ib-cr, qacE, oqxA, oqxB, fosA_5, mph(A), catB3, catA1, qnrB4, ARR-3, sul1, dfrA12*
**NDM-Kpn-48**	25/08/22	147	>256, R	>1, R	>8, R	>8, R	>=16, R	16, R	>=4, R	>64/4, R	≤ 1/19, S	0.5, S	*aph(3')-VI, ant(3'')-Ia, aac(6')-Ib-cr, acc(6')-Ib, acc(3)-IIa, qac(E), oqxA, oqxB, fosA_5, ere(A), catB3, catA1, cmlA1, qnrS1, AAR-2, sul1, tet(A)*
**NDM-Kpn-49**	23/08/22	147	>256, R	>1, R	>8, R	>8, R	2, S	16, R	>1, R	>16/4, R	≤ 1/19, S	0.25, S	*aph(3')-VI, aadA1, aac(6')-Ib-cr, acc(3)-IIa, qac(E), oqxA, oqxB, fosA_5, qnrS1, AAR-3, sul1*
**NDM-Kpn-50**	06/09/22	11	>256, R	>2, R	>8, R	>8, R	>8, R	>16, R	>1, R	>64/4, R	>4/76, R	0.5, S	*aadA2, aph(3')-Ia, rmtB, aac(6')-Ib-cr, qacE, oqxA, oqxB, fosA_5, mph(A), catB3, catA1, qnrB4, ARR-3, sul1, dfrA12*
**NDM-Kpn-51**	06/09/22	147	>256, R	>2, R	>8, R	>8, R	>8, R	16, R	>1, R	>64/4, R	≤ 1/19, S	0.5, S	*aph(3')-VI, ant(3'')-Ia, aac(6')-Ib-cr, acc(6')-Ib, acc(3)-IIa, qac(E), oqxA, oqxB, fosA_5, ere(A), catB3, catA1, cmlA1, qnrS1, AAR-2, sul1, tet(A)*
**NDM-Kpn-52**	06/09/22	11	>256, R	>2, R	>8, R	>8, R	>8, R	>16, R	>1, R	>64/4, R	>4/76, R	0.5, S	*aadA2, aph(3')-I, rmtB, aac(6')-Ib-cr, qacE, oqxA, oqxB, fosA_5, mph(A), catB3, catA1, qnrB4, ARR-3, sul1, dfrA12*
**NDM-Kpn-53**	09/09/22	395	>256, R	>1, R	>8, R	>8, R	>4, R	>16, R	>1, R	>16/4, R	>4/76, R	1, S	*aph(3')-VI, armA, aac(6')-Ib-cr, qac(E), oqxA, oqxB, fosA_5, msr(E), mph(A), mph(E), catB3, catA1, qnrS1, sul1, sul2, dfrA5*
**NDM-Kpn-54**	20/09/22	15	>256, R	>1, R	>8, R	>8, R	>4, R	>16, R	>1, R	>16/4, R	≤ 1/19, S	1, S	*aac(6')-Ib3, rmtC, qac(E), oqxA, oqxB, fosA_5, sul1*
**NDM-Kpn-55**	25/11/22	147	>256, R	>1, R	>8, R	>8, R	>8, R	>16, R	>1, R	>64/4, R	>4/76, R	0.5, S	*aadA2, aac(6')-Ib-cr, rmtB, qacE, oqxA, oqxB, fosA_5, mph(A), erm(B), catB3, qnrS1, ARR-3, sul1, dfrA12*
**NDM-Kpn-56**	19/01/23	147	>256, R	>1, R	>8, R	>8, R	>4, R	>16, R	>1, R	>16/4, R	>4/76, R	1, S	*aph(3')-VI, aadA2, armA, qac(E), oqxA, oqxB, fosA_5, msr(E), mph(A), mph(E), sul1, tet(A), dfrA12*
**NDM-Kpn-57**	02/02/23	395	>256, R	>1, R	>8, R	>8, R	>4, R	>16, R	>1, R	>16/4, R	>4/76, R	1, S	*aph(3')-VI, aac(6')-Ib-cr, armA1, qac(E), oqxA1, oqxB1, fosA_3, msr(E), mph(A), mph(E), catB3, catA1, qnrS1, sul1, sul2, dfrA5*
**NDM-Kpn-58**	14/02/23	15	>256, R	>1, R	>8, R	>8, R	>4, R	>16, R	>1, R	>16/4, R	≤ 1/19, S	1, S	*aac(6')-Ib-3, rmtC, qac(E), oqxA, oqxB, fosA_3, sul1*
**NDM-Kpn-59**	21/02/23	147	>256, R	>1, R	>8, R	>8, R	>4, R	>16, R	>1, R	>64/4, R	>4/76, R	16, R	*aph(3')-VI, armA1, qac(E), oqxA1, oqxB1, fosA_5, msr(E), mph(A), mph(E), catB3, qnrS1, ARR-3, sul1, sul2, dfrA5*
**NDM-Kpn-60**	23/02/23	147	>256, R	>1, R	>8, R	>8, R	>4, R	>16, R	>1, R	>64/4, R	>4/76, R	1, S	*aph(3')-VI, aac(6')-Ib, ant(3'')-Ia, armA1, aac(6')-Ib-cr, qac(E), oqxA1, oqxB1, fosA_5, msr(E), mph(A), mph(E), catB3, qnrS1, ARR-3, sul1, sul2, dfrA5*
**NDM-Kpn-61**	01/03/23	147	>256, R	>1, R	>8, R	>8, R	>=16, R	32, R	>=4, R	>64/4, R	>4/76, R	8, R	*aph(3')-VI, aac(6')-Ib, ant(3'')-Ia, armA1, aac(6')-Ib-cr, qac(E), oqxA1, oqxB1, fosA_3, fosA_5, msr(E), mph(A), mph(E), catB3, qnrS1, ARR-3, sul1, sul2, dfrA5*

**NDM-Kpn-62**	01/03/23	11	>256, R	>1, R	>8, R	>8, R	>=32, R	16, R	>=8, R	>64/4, R	>4/76, R	1, S	*aac(6')-Ib-cr, aac(6')-Ib-1, acc(3)-IIa, oqxA1, oqxB1, fosA_3, catB3, dfrA14*
**NDM-Kpn-63**	19/05/23	11	>256, R	>1, R	>8, R	>8, R	>4, R	16, R	>1, R	>64/4, R	≤ 1/19, S	0.5, S	*aac(6')-Ib-1, aac(6')-Ib-cr, acc(3)-IIa, oqxA1, oqxB1, fosA_3, catB3, dfrA14*
**NDM-Kpn-64**	19/05/23	1805	>256, R	>1, R	>8, R	>8, R	>4, R	>16, R	1, R	>64/4, R	>4/76, R	0.5, S	*aph(3')-VI, armA1, qacE, oqxA1, oqxB1, fosA_3, msr(E), mph(A), mph(E), qnrS1, sul1, sul2, dfrA5*
**NDM-Kpn-65**	23/05/23	147	>256, R	>1, R	>8, R	>8, R	>4, R	>16, R	>1, R	>64/4, R	>4/76, R	0.5, S	*aph(3')-VI, aadA2, armA, qacE, oqxA1, oqxB1, fosA_5, msr(E), mph(A), mph(E), sul1, tet(A), dfrA12*
**NDM-Kpn-66**	29/05/23	147	>256, R	>1, R	>8, R	>8, R	>4, R	>16, R	>1, R	>16/4, R	>4/76, R	4, R	*aph(3')-VI, aac(6')-Ib-1, ant(3'')-Ia, armA1, aac(6')-Ib-cr, qacE, oqxA1, oqxB1, fosA_5, msr(E), mph(A), mph(E), catB3, qnrS1, ARR-3, sul1, sul2, dfrA5*
**NDM-Kpn-67**	29/05/23	147	>256, R	>1, R	>8, R	>8, R	>4, R	>16, R	>1, R	>16/4, R	>4/76, R	1, S	*aph(3')-VI, aac(6')-Ib-1, aadA1, armA1, aac(6')-Ib-cr, qacE, oqxA1, oqxB1, fosA_5, msr(E), mph(A), mph(E), catB3, qnrS1, ARR-3, sul1, sul2, dfrA5*
**NDM-Kpn-68**	29/05/23	147	>256, R	>1, R	>8, R	>8, R	>4, R	>16, R	>1, R	>16/4, R	>4/76, R	0.25, S	*aph(3')-VI, aac(6')-Ib-1, ant(3'')-Ia, armA1, aac(6')-Ib-cr, qacE, oqxA1, oqxB1, fosA_5, msr(E), mph(A), mph(E), catB3, qnrS1, ARR-3, sul1, sul2, dfrA5*
**NDM-Kpn-69**	29/05/23	147	>256, R	>1, R	>8, R	>8, R	2, S	8, S	>1, R	>16/4, R	>4/76, R	4, R	*aph(3')-VI, aac(6')-Ib-1, ant(3'')-Ia, aac(6')-Ib-cr, qacE, oqxA1, oqxB1, fosA_5, msr(E), mph(A), mph(E), catB3, qnrS1, ARR-3, sul1, sul2, dfrA5*
**NDM-Kpn-70**	29/05/23	11	>256, R	>1, R	>8, R	>8, R	>4, R	>8, R	>1, R	>16/4, R	≤ 1/19, S	1, S	*aac(6')-Ib-1, ant(3'')-Ia, aac(6')-Ib-cr, aac(3')-IIa, oqxA1, oqxB1, fosA 5, catB3, dfrA14*
**NDM-Kpn-71**	30/05/23	1805	>256, R	>1, R	>8, R	>8, R	>4, R	>16, R	1, R	>16/4, R	>4/76, R	1, S	*aph(3')-VI, armA1, qacE, oqxA1, oqxB1, fosA_3, msr(E), mph(A), mph(E), qnrS1, sul1, sul2, tet(D), dfrA5*
**NDM-Kpn-72**	06/06/23	147	>256, R	>2, R	>8, R	>8, R	>8, R	>16, R	>1, R	>128, R	>4/76, R	0.5, S	*aph(6')-Id, aadA2, aac(6')-Ib-cr, rmtB, aph(3')-Ib, qacE, oqxA1, oqxB1, fosA_5, mph(A), erm(B), catB3, qnrB1, sul1, sul2, dfrA12*
**NDM-Kpn-73**	09/06/23	11	>256, R	>1, R	>8, R	>8, R	>4, R	16, R	>1, R	>16/4, R	>4/76, R	1, S	*aac(6')-Ib-1, aac(6')-Ib-cr, aac(3')-IIa, oqxA1, oqxB1, fosA_5, catB3, dfrA14*
**NDM-Kpn-74**	19/06/23	147	>256, R	>1, R	>8, R	>8, R	>4, R	>16, R	>1, R	>64/4, R	>4/76, R	1, S	*aph(3')-VI, aadA2, armA1, qacE, oqxA1, oqxB1, fosA_5, msr(E), mph(A), mph(E), sul1, tet(A), dfrA12*
**NDM-Kpn-75**	04/07/23	147	>256, R	>1, R	>8, R	>8, R	2, S	16, R	1, R	>64/4, R	≤ 1/19, S	1, S	*aph(3')-VI, aac (6')-Ib-1, aadA1, aac (6')-Ib-cr, qacE, oqxA1, oqxB1, fosA 5, catB3, qnrS1, ARR-3, sul1*
**NDM-Kpn-76**	04/07/23	1805	>256, R	>1, R	>8, R	>8, R	>4, R	>16, R	>1, R	>16/4, R	>4/76, R	1, S	*aph(3')-VI, armA1, qacE, oqxA1, oqxB1, fosA_3, msr(E), mph(A), mph(E), qnrS1, sul1, sul2, tet(D), dfrA5*
**NDM-Kpn-77**	04/07/23	147	>256, R	>1, R	>8, R	>8, R	>4, R	>16, R	>1, R	>16/4, R	>4/76, R	1, S	*aph(3')-VI, aadA2, armA1, qacE, oqxA1, oqxB1, fosA_5, msr(E), mph(A), mph(E), sul1, tet(A), dfrA12*
**NDM-Kpn-78**	11/07/23	234	>256, R	>1, R	>8, R	>8, R	≤1, S	≤4, S	>1, R	>16/4, R	>4/76, R	1, S	*aph(6)-Id, aadA2, aph(3')-Ib, qacE, oqxA1, oqxB1, fosA_3, mph(A), catA2, qnrS1, sul1, sul2, dfrA12*
**NDM-Kpn-79**	26/07/23	1805	>256, R	>1, R	>8, R	>8, R	>4, R	>16, R	>1, R	64/4, R	>4/76, R	1, S	*aph(3')-VI, armA1, qacE, oqxA1, oqxB1, fosA_3, msr(E), mph(A), mph(E), qnrS1, sul1, sul2, tet(D), dfrA5*
**NDM-Kpn-80**	02/08/23	1117	>256, R	>1, R	>8, R	>8, R	>4, R	>16, R	>1, R	>16/4, R	>4/76, R	1, S	*ant(2'')-Ia, aadA1, armA, qacE, oqxA, oqxB, fosA3, fosA6, msr(E), mph(E), cmlA5, ARR-2, sul1, sul3, dfrA12*
**NDM-Kpn-81**	02/08/23	147	>8/4, R	>1, R	>8, R	>8, R	>=16, R	>16, R	>=4, R	>64/4, R	>4/76, R	1, S	*aph(3')-VI, aac (6')-Ib-1, ant(3'')-Ia, aac(6')-Ib-cr, aadA2, armA, qacE, oqxA1, oqxB1, fosA_5, msr(E), mph(A), mph(E), qnrS1, catB3, sul1, sul2, dfrA5*
**NDM-Kpn-82**	12/08/23	147	>8/4, R	>1, R	>8, R	>8, R	>4, R	>16, R	>1, R	>16/4, R	>4/76, R	1, S	*aph(3')-VI, aadA2, armA, qacE, oqxA1, oqxB1, fosA_5, msr(E), mph(A), mph(E), sul1, tet(A), dfrA12*
**NDM-Kpn-83**	16/08/23	147	>8/4, R	>1, R	>8, R	>8, R	>4, R	>16, R	>1, R	>64/4, R	>4/76, R	1, S	*aph(3')-VI, aadA2, armA, qacE, oqxA1, oqxB1, fosA_5, msr(E), mph(A), mph(E), sul1, tet(A), dfrA12*

**NDM-Kpn-84**	22/08/23	11	>8/4, R	>1, R	>8, R	>8, R	32, R	8, S	>8, R	>64/4, R	≤ 1/19, S	1, S	*aac(6')-Ib, aac(6')-Ib-cr, aac(3')-IIa, oqxA1, oqxB1, fosA_3, catB3, dfrA14*
**NDM-Kpn-85**	22/08/23	147	>8/4, R	>1, R	>8, R	>8, R	>4, R	>16, R	>1, R	>16/4, R	>4/76, R	1, S	*aph(3')-VI, aadA2, armA, qacE, oqxA1, oqxB1, fosA_5, msr(E), mph(A), mph(E), sul1, tet(A), dfrA12*
**NDM-Kpn-86**	04/09/23	147	>8/4, R	>1, R	>8, R	>8, R	>4, R	>16, R	>1, R	>64/4, R	>4/76, R	4, R	*aph(3')-VI, aac (6')-Ib-1, ant(3'')-Ia, aac(6')-Ib-cr, armA, qacE, oqxA1, oqxB1, fosA_5, msr(E), mph(A), mph(E), qnrS1, catB3, ARR-3, sul1, sul2, dfrA5*
**NDM-Kpn-87**	08/09/23	147	>8/4, R	>1, R	>8, R	>8, R	1, S	8, S	>8, R	>64/4, R	>4/76, R	1, S	*aph(3')-VI, aac (6')-Ib-1, ant(3'')-Ia, aac(6')-Ib-cr, qacE, oqxA1, oqxB1, fosA_5, mph(A), qnrS1, catB2, ARR-3, sul1, dfrA12*
**NDM-Kpn-88**	08/09/23	147	>8/4, R	>1, R	>8, R	>8, R	1, S	>16, R	>8, R	>64/4, R	>4/76, R	1, S	*aph(3')-VI, aph(3')-XV, aac (6')-Ib-1, ant(3'')-Ia, aac(6')-Ib-cr, qacE, oqxA1, oqxB1, fosA_5, mph(A), qnrS1, catB2, ARR-3, sul1, dfrA14*
**NDM-Kpn-89**	12/09/23	11	256/4 R	>1, R	>8, R	>8, R	>4, R	16, R	>1, R	>16/4, R	≤ 1/19, S	1, S	*ant(2'')-Ia, aac(6')-Ib, aph(3')-Ia, oqxA1, oqxB1, fosA_3, qnrB19, dfrA14*
**NDM-Kpn-90**	12/09/23	147	256/4 R	>1, R	>8, R	>8, R	2, S	16, R	>1, R	>16/4, R	>4/76, R	1, S	*aph(3')-VI, aph(3')-XV, aac (6')-Ib-1, ant(3'')-Ia, aac(6')-Ib-cr, qacE, oqxA1, oqxB1, fosA_5, mph(A), qnrS1, catB2, ARR-3, sul1, dfrA14*
**NDM-Kpn-91**	19/09/23	147	>16/4, R	>1, R	>8, R	>8, R	>4, R	>16, R	>1, R	>16/4, R	>4/76, R	1, S	*aph(3')-VI, aadA2, armA, qacE, oqxA1, oqxB1, fosA_5, msr(E), mph(A), mph(E), sul1, tet(A), dfrA12*
**NDM-Kpn-92**	20/09/23	1805	>=16, R	>1, R	>8, R	>8, R	>=16, R	>16, R	>1, R	>64/4, R	>4/76, R	1, S	*aph(3')-VI, armA, qacE, oqxA1, oqxB1, fosA_5, msr(E), mph(A), mph(E), sul1, sul2, tet(D), dfrA5*
**NDM-Kpn-93**	27/09/23	147	>8/4, R	>1, R	>8, R	>8, R	>4, R	16, R	>1, R	>16/4, R	<=1/19, S	0.5, S	*aph(3')-XV, aac (6')-Ib-1, ant(3'')-Ia, qacE, oqxA1, oqxB1, fosA_5, mph(A), qnrS1, catB2, sul1, dfrA14*
**NDM-Kpn-94**	28/09/23	147	>128, R	>8, R	>8, R	>8, R	>=16, R	32, R	>=4, R	>64/4, R	>=320, R	0.5, S	*aph(3')-VI, armA, aac(6')-Ib-cr , qacE, oqxA1, oqxB1, fosA_5, msr(E), mph(A), mph(E), qnrS1, catB3, ARR-3, sul1, sul2, dfrA5*
**NDM-Kpn-95**	03/10/23	1805	>128, R	>8, R	>8, R	>8, R	32, R	>16, R	1, R	>64/4, R	>4/76, R	1, S	*aph(3')-VI, armA, qacE, oqxA1, oqxB1, fosA_3, msr(E), mph(A), mph(E), sul1, sul2, tet(D), dfrA5*
**NDM-Kpn-96**	06/10/23	147	>8/4, R	>1, R	>8, R	>8, R	>4, R	>16, R	>1, R	>16/4, R	>4/76, R	1, S	*aph(3')-VI, aadA2, armA, qacE, oqxA1, oqxB1, fosA_5, msr(E), mph(A), mph(E), sul1, tet(A), dfrA12*
**NDM-Kpn-97**	18/10/23	3299	>8/4, R	>2, R	>8, R	>8, R	>4, R	>32, R	>1, R	>16/4, R	>4/76, R	1, S	*aac(6')-Ib3, ant(3'')-Ia, aph(3')-Ia, aph(3')-Ib, rmtC, qac(E), oqxA, oqxB, fosA_5, erm(B), mph(A), catA2, qnrS1, sul1, sul2, tet(A), dfrA12*
**NDM-Kpn-98**	24/10/23	11	>8/4, R	>1, R	>8, R	>8, R	>=16, R	>16, R	>=4, R	>64/4, R	≤ 1/19, S	0.5, S	*ant(2'')-Ia, aac(6')-Ib, aph(3')-Ia, aac(6')-Ib-cr, oqxA1, oqxB1, fosA_5, catB3, qnrB19, dfrA14*
**NDM-Kpn-99**	10/11/23	11	256/4, R	>1, R	>8, R	>8, R	>4, R	>16, R	>1, R	>16/4, R	≤ 1/19, S	1, S	*aac(6')-Ib, aac(6')-Ib-cr, oqxA1, oqxB1, fosA_3, catB3, dfrA14*
**NDM-Kpn-100**	15/11/23	4081	>8/4, R	>1, R	>8, R	>8, R	1, S	≤1, S	>1, R	>64/4, R	>4/76, R	1, S	*aadA2, qacE, oqxA, oqxB, fosA_6, sul1, dfrA12*
**NDM-Kpn-101**	15/11/23	395	>8/4, R	>1, R	>8, R	>8, R	>32, R	>32, R	>8, R	>64/4, R	>4/76, R	1, S	*aac(6')-Ib-cr, rmtC, qacE, oqxA1, oqxB1, fosA_3, catB3, qnrS1, tet(A), dfrA1*
**NDM-Kpn-102**	15/11/23	147	>8/4, R	>2, R	>8, R	>8, R	<=1, S	8, S	>1, R	>64/4, R	>4/76, R	0.5, S	*aph(3')-VI, aac (6')-Ib-1, ant(3'')-Ia, aac(6')-Ib-cr, qacE, oqxA1, oqxB1, fosA_5, mph(A), qnrS1, catB2, ARR-3, sul1, dfrA14*
**NDM-Kpn-103**	24/11/23	147	256/4, R	>1, R	>8, R	>8, R	>4, R	>16, R	>1, R	>16/4 R	>4/76, R	1, S	*aph(3')-VI, aadA2, armA, qacE, oqxA1, oqxB1, fosA_5, msr(E), mph(A), mph(E), sul1, tet(A), dfrA12*
**NDM-Kpn-104**	24/11/23	11	>8/4, R	>1, R	>8, R	>8, R	>4, R	16, R	>1, R	>16/4 R	≤ 1/19, S	1, S	*aac(6')-Ib, aac(6')-Ib-cr, oqxA1, oqxB1, fosA_3, catB3, dfrA14*
**NDM-Kpn-105**	24/11/23	1805	>8/4, R	>1, R	>8, R	>8, R	>4, R	>16, R	1, R	>16/4 R	>4/76, R	1, S	*aph(3')-VI, armA, msr(E), mph(A), mph(E), qnrS1, sul1, sul2, tet(D), dfrA5*
**NDM-Kpn-106**	24/11/23	11	>8/4, R	>1, R	>8, R	>8, R	>4, R	16, R	>1, R	>16/4 R	≤ 1/19, S	1, S	*aac(6')-Ib, aac(6')-Ib-cr, aac(3')-IIa, oqxA1, oqxB1, fosA_3, catB3, dfrA14*

**NDM-Kpn-107**	24/11/23	383	>8/4, R	>1, R	>8, R	>8, R	2, S	8, S	>1, R	>16/4 R	≤ 1/19, S	1, S	*aac(6')-Ib, aph(3')-Ia, aph(3')-Ib, aph(3')-VI, aph(6')-Id, aadA1, oqxA1, oqxB1, fosA_3, catB3, mph(A), catA1, qnrS1, sul2, tet(A)*
**NDM-Kpn-108**	24/11/23	11	>8/4, R	>1, R	>8, R	>8, R	>4, R	16, R	>1, R	>16/4 R	≤ 1/19, S	1, S	*ant(2'')-Ia, aac(6')-Ib, aph(3')-Ia, aac(6')-Ib-cr, aac(3')-IIa, oqxA1, oqxB1, fosA_3, catB3, qnrB19, dfrA14*
**NDM-Kpn-109**	24/11/23	11	>8/4, R	>1, R	>8, R	>8, R	2, S	16, R	>1, R	>16/4, R	≤ 1/19, S	1, S	*aac(6')-Ib, aac(6')-Ib-cr, oqxA1, oqxB1, fosA_3, catB3, dfrA14*
**NDM-Kpn-110**	24/11/23	11	>8/4, R	>1, R	>8, R	>8, R	2, S	16, R	>1, R	>16/4, R	≤ 1/19, S	1, S	*aac(6')-Ib, aac(6')-Ib-cr, oqxA1, oqxB1, fosA_3, catB3, dfrA14*
**NDM-Kpn-111**	24/11/23	147	>8/4, R	>1, R	>8, R	>8, R	2, S	16, R	>1, R	>16/4, R	≤ 1/19, S	1, S	*aac(6')-Ib, aph(3')-VI, aadA1, aac(6')-Ib-cr, qacE, oqxA1, oqxB1, fosA_5, catB3, qnrS1, ARR-3, sul1*
**NDM-Kpn-112**	24/11/23	147	>8/4, R	>1, R	>8, R	>8, R	>4, R	16, R	>1, R	>16/4, R	>4/76, R	1, S	*aph(6')-Id, aac(6')-Ib, aac(3')-II, aph(3')-VI, aph(3'')-Ib, aadA1, qacE, oqxA1, oqxB1, fosA_5, mph(A), floR, catA1, qnrS1, sul1, sul2, tet(A), dfrA5*
**NDM-Kpn-113**	24/11/23	147	>8/4, R	>1, R	>8, R	>8, R	>4, R	>16, R	>1, R	>16/4, R	>4/76, R	1, S	*aph(3')-VI, aadA1, armA, qacE, oqxA1, fosA_5, msrE, mph(A), mph(E), sul1, tet(A), dfrA12*
**NDM-Kpn-114**	24/11/23	11	>8/4, R	>1, R	>8, R	>8, R	2, S	>8, R	>1, R	>16/4, R	≤ 1/19, S	1, S	*aac(6')-Ib, aac(6')-Ib-cr, oqxA1, oqxB1, fosA_3, catB3, dfrA14*
**NDM-Kpn-115**	24/11/23	11	>8/4, R	>1, R	>8, R	>8, R	>4, R	16, R	>1, R	>16/4, R	≤ 1/19, S	1, S	*ant(2'')-Ia, aac(6')-Ib, aph(3')-Ia, aac(6')-Ib-cr, aac(3')-IIa, oqxA1, oqxB1, fosA_3, catB3, qnrB19, dfrA14*
**NDM-Kpn-116**	24/11/23	147	>8/4, R	>1, R	>8, R	>8, R	2, S	>16, R	>1, R	>64/4, R	>4/76, R	0.5, S	*aph(3')-VI, aac (6')-Ib-1, ant(3'')-Ia, aac(6')-Ib-cr, qacE, oqxA1, oqxB1, fosA_5, mph(A), qnrS1, catB2, ARR-3, sul1, dfrA14*
**NDM-Kpn-117**	20/11/23	14	>8/4, R	>1, R	>8, R	>8, R	<=1, S	8, S	>1, R	>64/4, R	>4/76, R	0.25, S	*aac(6')-II, aph(3')-VI, qacE, oqxA1, oqxB1, fosA_3, qnrS1, sul1*
**NDM-Kpn-118**	29/11/23	11	>8/4, R	>1, R	>8, R	>8, R	>4, R	16, R	>1, R	>16/4, R	>4/76, R	1, S	*aac(6')-Ib, aac(6')-Ib-cr, oqxA1, oqxB1, fosA_3, catB3, dfrA14*
**NDM-Kpn-119**	01/12/23	147	>8/4, R	>1, R	>8, R	>8, R	>4, R	>16, R	>1, R	>64/4, R	>4/76, R	0.25, S	*aph(3')-VI, aph(3)-Ia, armA, aac(6')-Ib-cr, qacE, oqxA1, oqxB1, fosA_5, mph(A), mph(E), qnrS1, catB3, ARR-3, sul1, sul2, dfrA14*
**NDM-Kpn-120**	12/12/23	147	256/4, R	>1, R	>8, R	>8, R	>4, R	>16, R	>1, R	>16/4, R	>4/76, R	4, R	*aph(3')-VI, aac(6')-Ib, ant(3'')-Ia, armA, aac(6')-Ib-cr, qacE, oqxA1, oqxB1, fosA_5, msrE, mph(A), mph(E), catB3, qnrS1, ARR-3, sul1, sul2, dfrA5*
**NDM-Kpn-121**	07/12/23	147	256/4, R	>1, R	>8, R	>8, R	>4, R	>8, R	>1, R	>16/4, R	>4/76, R	1, S	*aph(3')-VI, aadA2, armA, qacE, oqxA1, oqxB1, fosA_5, msr(E), mph(A), mph(E), sul1, tet(A), dfrA12*
**NDM-Kpn-122**	14/12/23	395	>8/4, R	>1, R	>8, R	>8, R	>4, R	>16, R	>1, R	>16/4, R	>4/76, R	1, S	*aph(6')-Id, aph(3')-VI, aph(3')-VIa, armA, aac(6')-Ib-cr , qacE, oqxA1, oqxB1, fosA_3, msr(E), mph(A), mph(E), qnrS1, catB3, catA1, sul1, sul2, tet(A), dfrA5, dfrA1*
**NDM-Kpn-123**	15/12/23	147	>8/4, R	>1, R	>8, R	>8, R	>4, R	>16, R	>8, R	>64/4, R	>4/76, R	1, S	*aph(3')-VI, aac (6')-Ib-1, ant(3'')-Ia, aac(6')-Ib-cr, qacE, oqxA1, oqxB1, fosA_5, mph(A), qnrS1, catB2, ARR-3, sul1, dfrA14*
**NDM-Kpn-124**	20/12/23	6118	>8/4, R	>1, R	>8, R	>8, R	>4, R	>16, R	>1, R	>16/4, R	>4/76, R	1, S	*aac(6')-Ib, aph(3')-Ia, aph(3')-Ib, aph(3')-VI, aph(6')-Id, aadA1, armA, qacE, oqxA1, oqxB1, fosA_3, catB3, msr(E), mph(A), mph(E), catA1, qnrS1, sul1, sul2, tet(A), dfrA5*
**NDM-Kpn-125**	20/12/23	11	>8/4, R	>1, R	>8, R	>8, R	2, S	16, R	>1, R	>16/4, R	≤ 1/19, S	1, S	*aac(6')-Ib, aac(6')-Ib-cr, oqxA1, oqxB1, fosA_3, catB3, dfrA14*
**NDM-Kpn-126**	20/12/23	147	>8/4, R	>1, R	>8, R	>8, R	2, S	>16, R	>1, R	>16/4, R	>4/76, R	1, S	*aph(3')-VI, aadA2, armA, qacE, oqxA1, fosA_5, msr(E), mph(A), mph(E), sul1, tet(A), dfrA12*

[1]ST: Sequence Type; [2]CZA, ceftazidime/avibactam; ERT, ertapenem; IPM, imipenem; MEM, meropenem; GEN, gentamicin; AMK, amikacin; CIP, ciprofloxacin; TZP, piperacillin-tazobactam; SXT, trimethoprim-sulfametossazole; COL, colistin; S, susceptible; R, resistant.
